# Association of HIV and ART with cardiometabolic traits in sub-Saharan Africa: a systematic review and meta-analysis

**DOI:** 10.1093/ije/dyt198

**Published:** 2014-01-08

**Authors:** David G Dillon, Deepti Gurdasani, Johanna Riha, Kenneth Ekoru, Gershim Asiki, Billy N Mayanja, Naomi S Levitt, Nigel J Crowther, Moffat Nyirenda, Marina Njelekela, Kaushik Ramaiya, Ousman Nyan, Olanisun O Adewole, Kathryn Anastos, Livio Azzoni, W Henry Boom, Caterina Compostella, Joel A Dave, Halima Dawood, Christian Erikstrup, Carla M Fourie, Henrik Friis, Annamarie Kruger, John A Idoko, Chris T Longenecker, Suzanne Mbondi, Japheth E Mukaya, Eugene Mutimura, Chiratidzo E Ndhlovu, George Praygod, Eric W Pefura Yone, Mar Pujades-Rodriguez, Nyagosya Range, Mahmoud U Sani, Aletta E Schutte, Karen Sliwa, Phyllis C Tien, Este H Vorster, Corinna Walsh, Rutendo Zinyama, Fredirick Mashili, Eugene Sobngwi, Clement Adebamowo, Anatoli Kamali, Janet Seeley, Elizabeth H Young, Liam Smeeth, Ayesha A Motala, Pontiano Kaleebu, Manjinder S Sandhu

**Affiliations:** ^1^Department of Public Health and Primary Care, Institute of Public Health, University of Cambridge, Cambridge, UK, ^2^Genetic Epidemiology Group, Wellcome Trust Sanger Institute, Hinxton, Cambridge, UK, ^3^MRC/UVRI Uganda Research Unit on AIDS, Entebbe, Uganda, ^4^Division of Diabetic Medicine and Endocrinology, Department of Medicine, University of Cape Town, Cape Town, South Africa; Chronic Diseases Initiative in Africa, ^5^Department of Chemical Pathology, National Health Laboratory Service, University of the Witwatersrand Medical School, Johannesburg, South Africa, ^6^Malawi-Liverpool-Wellcome Trust Clinical Research Programme, Blantyre, Malawi, ^7^Department of Physiology, Muhimbili University of Health and Allied Sciences, Dar es Salaam, Tanzania, ^8^Department of Medicine, Muhimbili University of Health and Allied Sciences, Dar es Salaam, Tanzania, ^9^Royal Victoria Teaching Hospital, School of Medicine, University of The Gambia, Banjul, The Gambia, ^10^Department of Medicine, Obafemi Awolowo University, Ile Ife, Nigeria, ^11^Women's Equity in Access to Care &Treatment, Kigali, Rwanda, ^12^HIV-1 Immunopathogenesis Laboratory, Wistar Institute, Philadelphia, PA, ^13^Tuberculosis Research Unit, Department of Medicine, Case Western Reserve University, Cleveland, OH, ^14^Department of Medical and Surgical Sciences, University of Padua, Padua, Italy, ^15^Division of Diabetic Medicine and Endocrinology, Department of Medicine, University of Cape Town, Cape Town, South Africa, ^16^Infectious Diseases Unit, Department of Medicine, Grey's Hospital, Pietermaritzburg, South Africa, ^17^Department of Clinical Immunology, Aarhus University Hospital, Aarhus, Denmark, ^18^HART (Hypertension in Africa Research Team), North-West University, Potchefstroom, South Africa, ^19^Department of Nutrition, Exercise and Sports, Faculty of Science, University of Copenhagen, Copenhagen, Denmark, ^20^Africa Unit for Transdisciplinary Health Research (AUTHeR), North-West University, Potchefstroom, South Africa, ^21^Department of Medicine, Jos University Teaching Hospital, Jos, Nigeria, ^22^University Hospitals Case Medical Center, Cleveland, OH, ^23^German Development Cooperation (GTZ), Yaounde, Cameroon, ^24^Department of Medicine, Makerere University, Kampala, Uganda, ^25^Clinical Epidemiology Resource Training Centre, University of Zimbabwe College of Health Sciences, Harare, Zimbabwe, ^26^National Institute for Medical Research, Dar es Salaam, Tanzania, ^27^Chest Unit of Jamot Hospital, Yaounde, Cameroon, ^28^Epicentre, Médecins Sans Frontières, Paris, France, ^29^Clinical Epidemiology Group, Department of Epidemiology and Public Health, University College London, London, UK, ^30^Cardiology Unit, Department of Medicine, Aminu Kano Teaching Hospital, Kano, Nigeria, ^31^Soweto Cardiovascular Research Unit, Chris Hani Baragwanath Hospital, University of the Witwatersrand, Johannesburg, South Africa, ^32^Department of Medicine, University of California, San Francisco, CA, ^33^Faculty of Health Sciences, North-West University, Potchefstroom, South Africa, ^34^Department of Nutrition and Dietetics, University of the Free State, Bloemfontein, South Africa, ^35^Medical Research Council of Zimbabwe, Department of Medical Laboratory Sciences, University of Zimbabwe, Harare, Zimbabwe, ^36^Faculty of Medicine and Biomedical Sciences, University of Yaounde 1, Yaounde, Cameroon, ^37^Institute of Health and Society, University of Newcastle, Newcastle, UK, ^38^Institute of Human Virology, Abuja, Nigeria, ^39^Department of Epidemiology and Public Health, Institute of Human Virology and Greenebaum Cancer Center, University of Maryland School of Medicine, Baltimore, MD, ^40^Faculty of Epidemiology and Population Health, London School of Hygiene and Tropical Medicine, London, UK and ^41^Department of Diabetes and Endocrinology, Nelson R. Mandela School of Medicine, University of KwaZulu-Natal, Durban, South Africa

**Keywords:** HIV, ART, cardiometabolic disease, sub-Saharan Africa

## Abstract

**Background** Sub-Saharan Africa (SSA) has the highest burden of HIV in the world and a rising prevalence of cardiometabolic disease; however, the interrelationship between HIV, antiretroviral therapy (ART) and cardiometabolic traits is not well described in SSA populations.

**Methods** We conducted a systematic review and meta-analysis through MEDLINE and EMBASE (up to January 2012), as well as direct author contact. Eligible studies provided summary or individual-level data on one or more of the following traits in HIV+ and HIV-, or ART+ and ART- subgroups in SSA: body mass index (BMI), systolic blood pressure (SBP), diastolic blood pressure (DBP), high-density lipoprotein (HDL), low-density lipoprotein (LDL), triglycerides (TGs) and fasting blood glucose (FBG) or glycated hemoglobin (HbA1c). Information was synthesized under a random-effects model and the primary outcomes were the standardized mean differences (SMD) of the specified traits between subgroups of participants.

**Results** Data were obtained from 49 published and 3 unpublished studies which reported on 29 755 individuals. HIV infection was associated with higher TGs [SMD, 0.26; 95% confidence interval (CI), 0.08 to 0.44] and lower HDL (SMD, −0.59; 95% CI, −0.86 to −0.31), BMI (SMD, −0.32; 95% CI, −0.45 to −0.18), SBP (SMD, −0.40; 95% CI, −0.55 to −0.25) and DBP (SMD, −0.34; 95% CI, −0.51 to −0.17). Among HIV+ individuals, ART use was associated with higher LDL (SMD, 0.43; 95% CI, 0.14 to 0.72) and HDL (SMD, 0.39; 95% CI, 0.11 to 0.66), and lower HbA1c (SMD, −0.34; 95% CI, −0.62 to −0.06). Fully adjusted estimates from analyses of individual participant data were consistent with meta-analysis of summary estimates for most traits.

**Conclusions** Broadly consistent with results from populations of European descent, these results suggest differences in cardiometabolic traits between HIV-infected and uninfected individuals in SSA, which might be modified by ART use. In a region with the highest burden of HIV, it will be important to clarify these findings to reliably assess the need for monitoring and managing cardiometabolic risk in HIV-infected populations in SSA.

## Introduction

Sub-Saharan Africa (SSA) has the highest burden of HIV in the world, with approximately 22.9 million prevalent cases and 1.9 million new infections recorded in 2010.^1^ The estimated 1.3 million people who died of HIV-related illnesses in SSA in 2009 comprised 72% of the global mortality attributable to the epidemic.^2^ Anti-retroviral therapy (ART) coverage in this region has rapidly increased over the past decade, with 49% of eligible cases receiving treatment in 2010.^1^ Expanding use of ART has led to a notable decline in HIV-associated morbidity and death in SSA.^3^ As life expectancy among HIV-infected people improves, it is crucial to understand the long-term impact of HIV and its treatment in this region.^4^ Parallel to the changing landscape of HIV care, the burden of cardiometabolic diseases in SSA is increasing,^5^ with expected deaths attributable to cardiovascular disease projected to double to 2.4 million in 2030 relative to reports from 2000.^6^ These data suggest that cardiometabolic diseases will become a major health problem in SSA, competing with infectious diseases for limited health resources.^7–9^

Several studies in populations of European descent suggest that HIV infection and ART are independently associated with an increased risk of cardiometabolic disease, including cardiovascular disease, dyslipidaemia and type 2 diabetes (T2D).^10–13^ However, findings appear to be inconsistent even within these studies, and the true direction and magnitude of these associations remain uncertain. A large prospective study reported a 26% relative increase in the rate of myocardial infarction (MI) per year of ART exposure during the first 4–6 years of use.^14^ In 2003, to address possibly increased cardiometabolic risk in this group, the HIV Medicine Association of the Infectious Disease Society of America and the Adult AIDS Clinical Trials Group published guidelines specifically for management of dyslipidaemia in HIV-infected individuals.^15^ However, these guidelines were primarily based on evidence from European studies and have not been widely implemented in SSA.^16^

Importantly, there is some evidence to suggest that there may be differences in cardiometabolic risk profiles in people of African descent compared with people of European descent,^17–20^ implying that the aetiology of cardiometabolic disease, and the distribution and spectrum of risk factors, might differ in African populations. Examples include the differential tobacco usage patterns in SSA compared with other regions, as well as differences in alcohol consumption patterns in populations of African descent. Furthermore, it has been reported that the predominant virus strains responsible for HIV infection in SSA are HIV-1, group M (major) subtypes A and C,^21^ which differ as much as 30% in their genomes from HIV-1 subtype B, responsible for the infections in North America and Europe.^21^^,^^22^ The clinical consequences of these subtype differences are, as yet, unclear. Additionally, there is precedent for differences in the efficacy of infectious disease treatments in individuals of African descent, such as that seen in interferon treatment for chronic hepatitis C.^23^ These potential differences in HIV and ART associations with cardiometabolic traits, if any, have not been reliably clarified.

In this context, it is important to examine possible associations between HIV infection, ART treatment and cardiometabolic traits in SSA. Assessing these associations will help inform and guide future research and public health responses in the region. We therefore conducted a systematic review and meta-analysis of published and unpublished data to assess these associations in SSA.

## Methods

### Search strategy and identification of studies

This systematic review was conducted and reported in accordance with the PRISMA guidelines. This study focused on differences in cardiometabolic traits between HIV-infected and uninfected individuals, and between those receiving and not receiving treatment. A group of eight commonly accepted cardiometabolic traits were selected a priori for inclusion in this analysis: body mass index (BMI), systolic blood pressure (SBP), diastolic blood pressure (DBP), serum high-density lipoprotein cholesterol (HDL), serum low-density lipoprotein cholesterol (LDL), triglycerides (TGs), fasting blood glucose(FBG) and glycated haemoglobin (HbA1c). We did not examine lipodystrophy as a risk factor due to the marked variability in assessment criteria in the literature. Using a structured search strategy (Supplementary Figures 1–2, available as Supplementary data at *IJE* online), PUBMED and EMBASE databases were queried for articles written in English before the 1 January 2012. Published abstracts were reviewed and assessed for inclusion in the study. Those meeting the following inclusion criteria were listed for full text review (Box 1): described data on the relevant cardiometabolic traits in comparable HIV+ and HIV- populations, or comparable ART+ and ART naive groups; and included adult (aged 18 years or over) Black participants based in SSA, as defined by the WHO African region.^24^ Comparability between groups was defined as data collection using similar study procedures for both individuals infected and those uninfected with HIV, or ART users and nonusers. Two reviewers (D.G.D. and J.R.) independently assessed studies for eligibility. Consensus for eligibility between the two reviewers was >95%. Any discrepancies in eligible studies listed were resolved by consensus discussion. Studies not meeting both eligibility criteria were not included in the final review. We excluded case reports with fewer than five participants. Electronic searches were supplemented by cross-referencing of cited reference lists from retrieved articles and reviews.

**Box 1** Eligibility criteria for inclusion in the systematic review

**Table:** 

	Inclusion criteria
Population	A population or cohort consisting of adult Black participants based in sub-Saharan Africa, as defined by the World Health Organization African region Consists of comparable HIV+ and HIV− populations or comparable ART+ and ART− naive groups
Outcome	Presents data on at least one of the following: body mass index, systolic blood pressure, diastolic blood pressure, serum high-density lipoprotein cholesterol, low-density lipoprotein cholesterol and triglycerides, fasting blood glucose or HbA1c

ART, antiretroviral therapy.

**Figure 1:**
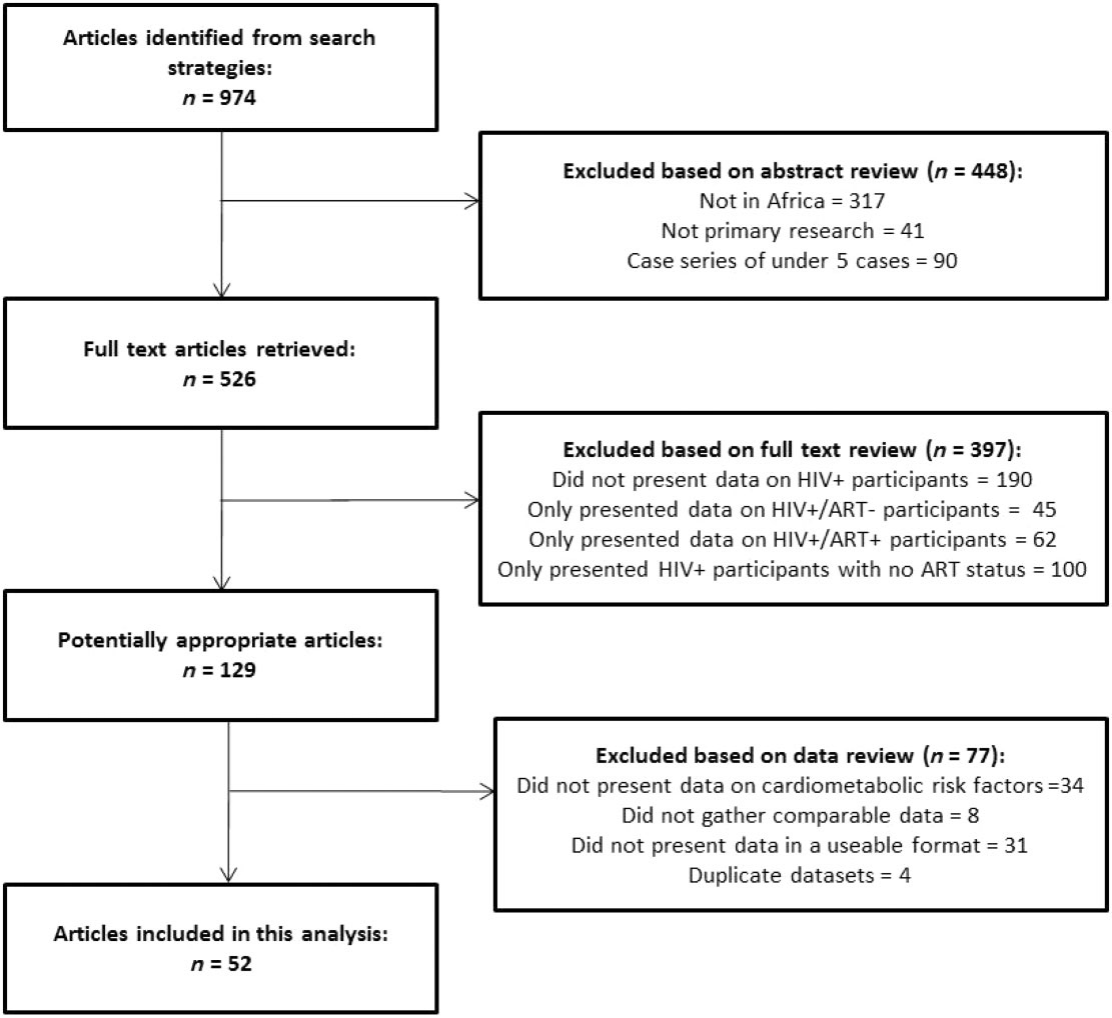
Study selection

Following full text review of all potentially eligible articles, those identified as fulfilling the inclusion criteria (Figure 1) were collated for analysis. We contacted the corresponding authors of all eligible articles, inviting their participation in this study. We worked with these authors to confirm the accuracy of extracted published data and to obtain additional relevant unpublished data for this review. Responses were received from 69.7% of the contacted authors, of whom 68.4% agreed to collaborate on this meta-analysis. We received data from 85.0% of collaborating groups. All studies were reviewed and approved by their respective research ethics committees. Full details of the search strategy, all identified articles and reasons for exclusion if applicable can be found in Supplementary Figures 1–2 and Supplementary Table 1, available as Supplementary data at *IJE* online.

### Data abstraction and synthesis

Year, country, publication status (published/unpublished) and study type (cohort/case-control) were recorded for each study. The following data were extracted for relevant subgroups (HIV+, HIV−, ART+, ART−) within each study: number of individuals, mean age, sex distribution, means and SDs for pre-specified cardiometabolic traits, and fasting status at time of measurement (Supplementary Table 2, available as Supplementary data at *IJE* online).

HIV status was defined by classification in each individual study without alteration. HIV infection was considered irrespective of ART status, and individuals receiving ART were not excluded from this group. We defined ‘ART use’ as receipt of ART medication at the time of cardiometabolic trait measurement in the original report. Due to heterogeneous study designs and the frequent lack of specific ART-related data in non-ART-centric studies, no specific data were gathered on ART type, ART duration pre-measurement or calendar period during receipt of ART. In accordance with the International System of Units (SI), all cardiometabolic measurements were converted to mmol/l, %, mmHg or kg/m^2^, as appropriate.

### Individual-level participant data from the General Population Cohort Study

In order to explore the impact of residual confounding on our estimates, and to assess consistency between unadjusted estimates from summary-level data and fully adjusted estimates from individual-level data, we also analysed previously unpublished individual-level data from one of the studies included in the meta-analysis—the General Population Cohort (GPC) study. These individual-level analyses were performed on 5586 participants, comprising 18.8% of the total number of participants included in this meta-analysis.

The GPC study is a population-based cohort study of approximately 22 000 individuals living in rural south-west Uganda. This cohort was established in 1989 by the Medical Research Council Programme on AIDS in Uganda to assess trends in the prevalence and incidence of HIV infection in the population. Since then, an annual census is taken of the entire population to collect basic demographic information. From this census, consenting individuals are invited to take part in an interviewer-mediated questionnaire, to have their biophysical measurements taken and to have blood samples drawn for analysis. GPC participants found to be HIV-infected are invited to join the Rural Clinical Cohort for further follow-up. The Rural Clinical Cohort encompasses all consenting HIV+ participants within the GPC and gathers data on their health and disease progression, in addition to providing care and access to ART. Full details of the cohort structure, measurement techniques and the annual HIV survey have been published elsewhere.^25^^,^^26^

In the GPC, detailed individual-level data were collected on HIV status, ART, age, sex, BMI, lipid factors, blood pressure, HbA1c levels, education status, smoking and household-level clustering (Supplementary Table 3, available as Supplementary data at *IJE* online). This study was approved by the Science and Ethics Committee of the Uganda Virus Research Institute, the Uganda National Council for Science and Technology and the East of England-Cambridge South (formerly Cambridgeshire 4) NHS Research Ethics Committee UK.

### Statistical analysis

Because we anticipated heterogeneity among results of studies due to potential differences in underlying genetic susceptibility, health care infrastructure and monitoring of chronic disease among individuals with and without HIV and those using ART, we used random-effects meta-analyses in our primary analyses. However, as results from random-effects meta-analyses may not always be conservative, we also compared random and fixed-effects estimates. We examined standardized mean difference (SMD)^27^ between relevant groups (HIV+, HIV−, ART+, ART−) as the primary measure of association for each trait for ease of interpretation. This summary measure allows the reader to compare differences in disparate cardiometabolic traits on a single scale, and comprehend these differences without an underlying knowledge of the normal values and distribution of the traits in question. The *I^2^* statistic was used to assess heterogeneity between studies.^28^

We initially explored potential sources of heterogeneity through the visual inspection of forest and Galbraith plots. Meta-regression and stratified analysis approaches were then used to assess the contribution of study-level variables to heterogeneity in summary estimates. Variables assessed were: study type (cohort/case-control), study size, date of publication, study location, publication status (published/unpublished), mean participant BMI, mean participant age, sex distribution, mean difference in BMI between groups, mean age difference between groups, and proportion of HIV-infected individuals on ART in each study (for comparisons between HIV-infected and uninfected individuals). For evaluation of heterogeneity by study location, studies were initially grouped according to UN geographical sub-areas as follows: East Africa, Central Africa, West Africa and Southern Africa. However, as data gathered from West and Central African regions were limited, these were collapsed for further analysis. Factors were identified as contributing to between-study heterogeneity, when a substantial reduction in heterogeneity was observed on adjustment for the factor in meta-regression. Heterogeneity resulting from differing ART drug class could not be explored because of the small number of studies that reported this information. Furthermore, we could not explore heterogeneity by participant fasting status, as a large proportion of studies did not report status during blood draw for lipid traits and all studies reporting glucose measurements were on fasted individuals. We also sought to systematically explore the potential impact of outliers on estimates from meta-analysis for each by evaluating the stability of meta-analysed SMD estimates to sequential exclusion of single studies.

In order to assess consistency between estimates from adjusted individual-level data and unadjusted summary data, we carried out individual participant data analysis on a subset of the meta-analytical data using the GPC study. We calculated SMD estimates for the differences in cardiometabolic traits associated with HIV infection and ART use, adjusted for age, sex, BMI, education level, smoking status and ART use (among HIV-infected individuals), using linear mixed-effects models, including random effects for data clustering at household and village levels. Age and BMI were added as continuous variables whereas sex, education level, smoking status and ART use were all added as categorical variables. All analyses were conducted in Stata version 11.0.

## Results

**Figure 2:**
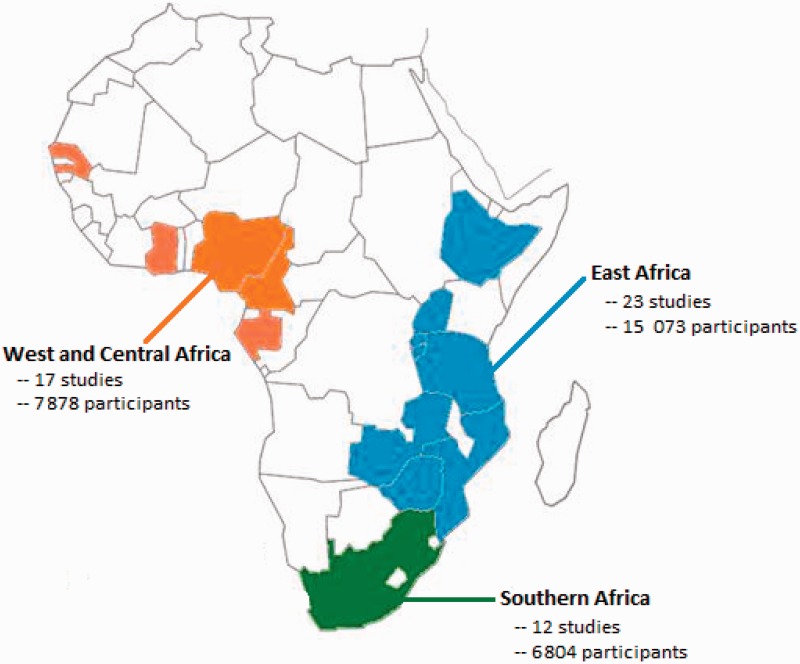
Countries contributing data, by region

We analysed 52^29–77^ datasets from 14 countries (Figure 2), providing study-level data on 29 755 participants (23 119 from previously published studies and 6636 participants from unpublished studies; Unpublished data acquired from personal communications with C. Fourie, A. Schutte, and the MRC/UVRI; Table 1). Studies were broadly distributed across the three regions in SSA, with more participants from East Africa than Southern Africa or West & Central Africa (Table 1). Of these 52 studies, nine were conducted among HIV and tuberculosis co-infected patients, two among malnourished populations and two among pregnant women. None of these study-level factors explained an appreciable portion of between study heterogeneity in meta-regression analyses (Table 2).

**Table 1:** Characteristics of included studies, by region

					**Number of participants by exposure group**			
	**Number of studies per region**	**Number of previously published participants**	**Number of unpublished participants**	**Total number of participants**	HIV+	HIV−	ART+	ART−
East Africa	23	9487	5586	15 073	6064	9009	1120	2674
West and Central Africa	17	7878	0	7878	4422	3456	622	648
Southern Africa	12	5754	1050	6804	2271	4533	600	906
Total	52	23 119	6636	29 755	12 757	16 998	4342	4228

	**Number of participants with data on each risk factor**							
	TG	HDL	LDL	BMI	SBP	DBP	Fasting glucose	HbA1c
East Africa	7791	7772	7777	14 315	6147	6146	459	5551
West and Central Africa	1627	1627	1627	6623	726	726	335	208
Southern Africa	6031	5581	5529	6602	6336	6339	4286	305
Total	15 449	14 980	14 933	27 540	13 209	13 211	5080	6064

BMI, body mass index; TGs, triglycerides; LDL, low-density lipoprotein cholesterol; HDL, high-density lipoprotein cholesterol; SBP, systolic blood pressure; DBP, diastolic blood pressure; HbA1c, glycated haemoglobin; ART, antiretroviral therapy.

**Table 2:** I^2^-values for residual heterogeneity after meta-regression incorporating study level characteristics

	Unadjusted	Study type	Study size	Date of study	Location	Publication status	Tuberculosis co-infection	Pregnant participants	Malnourished participants	Mean study BMI	Mean study age	Study sex distribution	Mean BMI difference between HIV+/HIV− or ART+/ART−	Mean age difference between HIV+/HIV− or ART+/ART−	Proportion of HIV+ participants on ART
**HIV associations**															
BMI	93.4 (36)	93.4/93.3 * (36)	93.4/93.6 (36)	93.4/93.3 (36)	93.4/92.8 (36)	93.4/93.3 (36)	93.4/92.1 (36)	93.4/93.1 (36)	93.4/93.4 (36)	93.4/93.2 (36)	95.1/95.4 (18)	95.3/95.1 (25)	N/A	95.1/95.2 (18)	94.9/94.9 (12)
TGs	91.6 (15)	91.6/87.6 * (15)	91.6/92.1 (15)	91.6/90.5 (15)	91.6/90.1 (15)	91.6/92.1 (15)	–	–	–	82.4/72.7 (13)	65.5/70.8 (6)	92.4/92.9 (12)	82.4/83.8 (13)	65.5/71.4 (6)	91.6/95.5 (10)
LDL	91.1 (14)	91.1/91.3 (14)	91.1/91.8 (14)	91.1/91.8 (14)	91.1/91.2 (14)	91.1/91.7 (14)	–	–	–	92.3/92.5 (12)	18.3/0.00 (6)	90.0/90.9 (11)	92.3/93.0 (12)	18.3/34.1 (6)	91.9/90.2 (10)
HDL	96.1 (14)	96.1/96.3 (14)	96.1/94.8 (14)	96.1/96.4 (14)	96.1/96.2 (14)	96.1/95.0 (14)	–	–	–	95.7/95.9 (12)	93.6/92.7 (6)	96.6/96.2 (11)	95.7/95.8 (12)	93.6/7.3 *** (6)	97.3/97.0 (10)
SBP	84.1 (15)	84.1/82.8 (15)	84.1/82.9 (15)	84.1/82.7 * (15)	84.1/84.6 (15)	84.1/78.7 (15)	84.1/84.1 (15)	84.1/85.1 (15)	–	86.1/87.3 (13)	51.7/56.5 (8)	78.3/76.6 (11)	86.1/86.9 (13)	51.7/53.0 (8)	76.6/20.2 (4)
DBP	87.6 (15)	87.6/81.3 * (15)	87.6/84.4 (15)	87.6/86.3 (15)	87.6/88.1 (15)	87.6/80.3 (15)	87.6/88.7 (15)	87.6/89.1 (15)	–	88.3/89.1 (13)	76.7/80.0 (8)	87.7/88.6 (11)	88.3/83.6 * (13)	76.7/77.1 (8)	95.2/96.8 (4)
Glucose	98.5 (6)	98.5/96.5 (6)	98.5/98.7 (6)	98.5/98.8 (6)	98.5/98.7 (6)	98.5/98.7 (6)	–	–	–	98.8/99.0 (5)	–	98.9/96.9 (4)	98.8/98.7 (5)	–	99.3/99.0 (3)
HbA1c	82.5 (3)	–	–	–	–	–	–	–	–	–	–	–	–	–	–
**ART associations**															
BMI	91.0 (13)	91.0/88.2 * (13)	91.0/91.4 (13)	91.0/91.7 (13)	91.0/91.3 (13)	91.0/90.8 (13)	91.0/92.0 (13)	–	91.0/91.9 (13)	91.4/91.2 (12)	76.7/79.6 (4)	95.7/96.5 (6)	N/A	76.7/48.7 (4)	N/A
TGs	65.7 (10)	65.7/69.5 (10)	65.7/68.9 (10)	65.7/69.5 (10)	65.7/25.4 * (10)	65.7/69.5 (10)	–	–	–	69.8/67.2 (7)	50.2/61.8 (4)	0.0/0.0 (3)	69.8/74.1 (7)	50.2/37.3 (4)	N/A
LDL	93.4 (10)	93.4/91.4 (10)	93.4/92.5 (10)	93.4/93.5 (10)	93.4/94.0 (10)	93.4/93.8 (10)	–	–	–	89.2/89.9 (7)	70.9/63.4 (4)	92.8/88.9 (3)	89.2/83.7 * (7)	70.9/69.7 (4)	N/A
HDL	92.9 (10)	92.9/92.3 (10)	92.9/89.2 * (10)	92.9/93.7 (10)	92.9/93.1 (10)	92.9/93.7 (10)	–	–	–	85.3/86.6 (7)	93.6/95.3 (4)	93.8/0.00 (3)	85.3/85.6 (7)	93.6/89.7 (4)	N/A
SBP	83.4 (6)	83.4/71.7 (6)	83.4/59.6 (6)	83.4/85.3 (6)	83.4/84.3 (6)	83.4/86.6 (6)	83.4/84.7 (6)	–	–	83.4/84.1 (6)	–	–	83.4/81.2 (6)	–	N/A
DBP	64.6 (6)	64.6/55.7 (6)	64.6/28.7 (6)	64.6/71.3 (6)	64.6/68.6 (6)	64.6/71.6 (6)	64.6/70.0 (6)	–	–	64.6/67.7 (6)	–	–	64.6/61.6 (6)	–	N/A
Glucose	90.4 (5)	90.4/92.3 (5)	90.4/85.5 (5)	90.4/91.6 (5)	90.4/85.7 (5)	90.4/92.8 (5)	–	–	–	90.4/85.2 (5)	–	–	90.4/91.8 (5)	–	N/A
HbA1c	56.9 (2)	–	–	–	–	–	–	–	–	–	–	–	–	–	N/A

All values presented as I-squared percent without addition of the study level characteristic/I-squared percent with the addition of the study level characteristic (number of studies with relevant data).

N/A, not applicable; –, insufficient information; BMI, body mass index; TGs, triglycerides; LDL, low-density lipoprotein cholesterol; HDL, high-density lipoprotein cholesterol; SBP, systolic blood pressure; DBP, diastolic blood pressure; HbA1c, glycated haemoglobin; ART, antiretroviral therapy.

*P-value ≤ 0.05; **P-value ≤ 0.01; ***P-value ≤ 0.001.

### HIV and cardiometabolic traits

**Figure 3:**
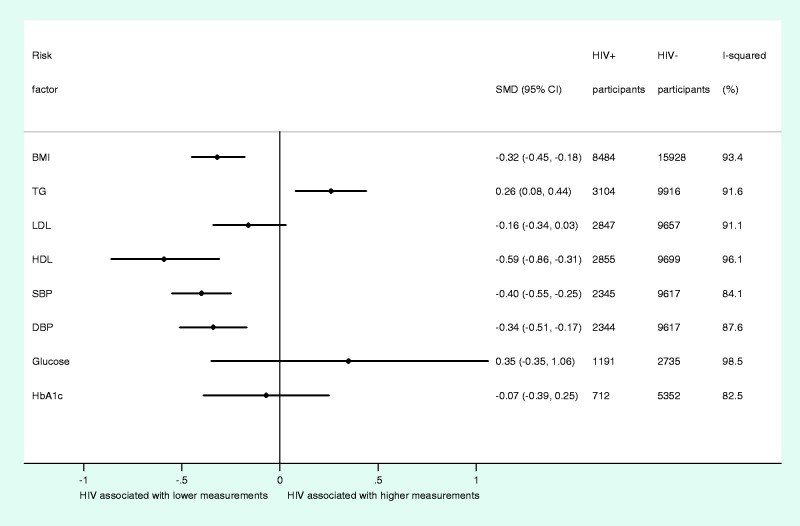
Summary of overall estimates from random-effects meta-analyses of associations between HIV and individual cardiometabolic risk factors. SMD, standardized mean difference; CI, confidence interval; BMI, body mass index; TGs, triglycerides; LDL, low-density lipoprotein cholesterol; HDL, high-density lipoprotein cholesterol; SBP, systolic blood pressure; DBP, diastolic blood pressure; HbA1c, glycated haemoglobin

In this meta-analysis of summary data from up to 29 755 study participants, we found that HIV infection was associated with lower mean BMI (SMD, −0.32; 95% CI, −0.45 to −0.18) (Figure 3). For blood lipids, HIV infection was associated with higher mean TG levels (SMD, 0.26; 95% CI, 0.08 to 0.44) and lower mean HDL levels (SMD, −0.59; 95% CI, −0.86 to −0.31), whereas no marked difference in mean LDL was observed between HIV− infected and uninfected individuals (SMD, −0.16; 95% CI, −0.34 to 0.03). HIV infection was also associated with lower DBP (SMD, −0.34; 95% CI, −0.51 to −0.17) and SBP (SMD, −0.40; 95% CI, −0.55 to −0.25) (Figure 3). Based on summary data from up to 6064 study participants, we did not find any evidence of association between HIV infection and fasting blood glucose or HbA1c (Figure 3). Study-level and combined summary estimates for each trait are illustrated in Supplementary Figures 3–10, available as Supplementary data at *IJE* online. Comparison of combined SMD estimates from fixed-effect and random-effect meta-analysis showed that the latter were consistently more conservative across all traits (Supplementary Figure 11, available as Supplementary data at *IJE* online).

We observed marked heterogeneity among combined SMDs for all traits (Figure 3). However, based on both stratified and meta-regression analyses, we found no consistent explanation for overall heterogeneity among studies for each trait, including study-level factors such as study size, year of study, publication status or study type (Table 2). Assessment of estimates stratified by study-level characteristics suggested that study location may have a modest impact on the magnitude of the association for some traits (Supplementary Figures 12–19, available as Supplementary data at *IJE* online). However, the addition of these variables into meta-regression did not affect heterogeneity estimates (Table 2 and Supplementary Figures 12–19, available as Supplementary data at *IJE* online). In addition, heterogeneity among SMDs in studies could not be explained by confounding factors measured at the study level (Table 2).

Visual inspection of forest and Galbraith plots suggested a variety of outlying studies for several cardiometabolic traits (Supplementary Figures 3–10 and 20–27, available as Supplementary data at *IJE* online), which may also impact on analyses exploring the determinants of heterogeneity. Sensitivity analyses examining the impact of extreme outlying studies on the combined SMDs of the cardiometabolic traits showed no material change in combined SMDs for traits found to be associated with HIV infection (Supplementary Tables 4–11, available as Supplementary data at *IJE* online). However, exclusion of a single outlying study led to associations, where there had previously been none, for two additional cardiometabolic traits—LDL and glucose. Table 3 describes the range of SMDs obtained for each trait after sequential exclusion of individual studies.

**Table 3:** Sensitivity analysis of the change in combined standardized mean difference estimates after sequential exclusion of single studies

	Combined estimate obtained before sequential exclusion			Instance in which exclusion of a single study produced a change in interpretation	
	SMD (95% CI)	I^2^	Range of SMDs obtained from sequential exclusion of individual studies	SMD (95% CI)	I^2^
**HIV associations**					
BMI	−0.32 (−0.45 to −0.18)	93.4%	−0.34 to −0.26	–	
TGs	0.26 (0.08 to 0.44)	91.6%	0.16 to 0.30	–	
LDL	−0.16 (−0.34 to 0.03)	91.1%	−0.27 to −0.11	Association observed after study exclusion^30^	
				−0.27 (−0.39 to −0.14)	79.4%
HDL	−0.59 (−0.86 to −0.31)	96.1%	−0.65 to −0.44	–	
SBP	−0.40 (−0.55 to −0.25)	84.1%	−0.44 to −0.37	–	
DBP	−0.34 (−0.51 to −0.17)	87.6%	−0.39 to −0.26	–	
Glucose	0.35 (−0.35 to 1.06)	98.5%	−0.14 to 0.50	Association observed after study exclusion^51^	
				−0.14 (−0.26 to −0.02)	43.0%
HbA1c	−0.07 (−0.39 to 0.25)	82.5%	−0.16 to 0.04	–	
**ART associations**					
BMI	0.12 (−0.11 to 0.34)	91.0%	0.02 to 0.15	–	
TGs	0.09 (−0.04 to 0.21)	65.7%	0.05 to 0.12	Association observed after study exclusion^74^	
				0.12 (0.00 to 0.24)	59.1%
LDL	0.43 (0.14 to 0.72)	93.4%	0.34 to 0.53	–	
HDL	0.39 (0.11 to 0.66)	92.9%	0.31 to 0.49	–	
SBP	0.05 (−0.19 to 0.28)	83.4%	−0.3 to 0.16	–	
DBP	0.06 (−0.10 to 0.22)	64.6%	0.00 to 0.16	Association observed after study exclusion^30^	
				0.16 (0.06 to 0.26)	8.8%
Glucose	−0.23 (−0.61 to 0.16)	90.4%	−0.34 to −0.04	–	
HbA1c	−0.34 (−0.62 to −0.6)	56.9%	−0.23 to −0.52	–	

–, combined SMD did not change statistical significance due to the sequential exclusion of any single study; CI, confidence interval; BMI, body mass index; TGs, triglycerides; LDL, low-density lipoprotein cholesterol; HDL, high-density lipoprotein cholesterol; SBP, systolic blood pressure; DBP, diastolic blood pressure; HbA1c, glycated haemoglobin; ART, antiretroviral therapy.

### ART and cardiometabolic traits

**Figure 4:**
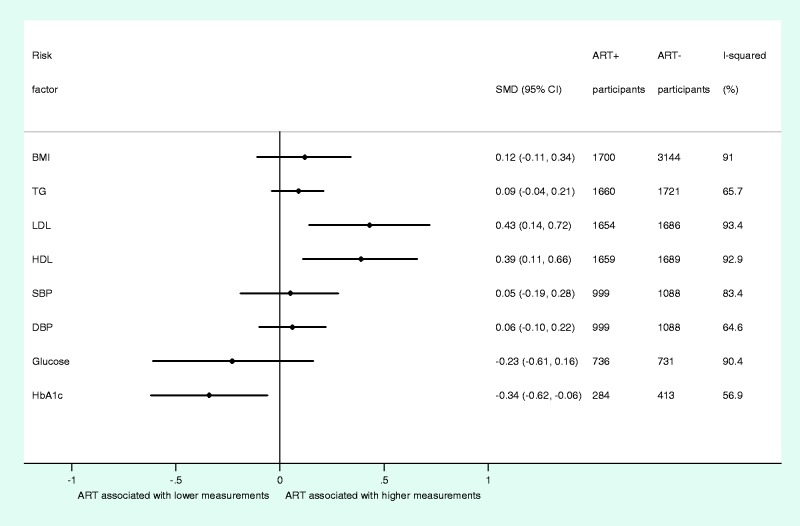
Summary of overall estimates from random-effects meta-analyses of associations between ART and individual cardiometabolic risk factors. SMD, standardized mean difference; CI, confidence interval; BMI, body mass index; TGs, triglycerides; LDL, low-density lipoprotein cholesterol; HDL, high-density lipoprotein cholesterol; SBP, systolic blood pressure; DBP, diastolic blood pressure; HbA1c, glycated haemoglobin; ART, antiretroviral therapy

In analyses based on up to 3348 HIV+ individuals, ART exposure was found to be associated with higher HDL (SMD, 0.39; 95% CI, 0.11 to 0.66) and LDL levels (SMD, 0.43; 95% CI, 0.14 to 0.72) and lower HbA1c levels (SMD, −0.34; 95% CI, −0.62 to −0.06) (Figure 4). By contrast, no appreciable differences were observed for BMI (SMD, 0.12; 95% CI, −0.11 to 0.34) or TGs (SMD, 0.09; 95% CI, −0.04 to 0.21) between ART users and non-users (Figure 4). Based on data from up to 2087 participants, we did not detect any association between ART use and SBP, DBP or fasting blood glucose (Figure 4). Individual study SMDs and combined estimates for each cardiometabolic trait are presented in Supplementary Figures 28–35, available as Supplementary data at *IJE* online. Estimates from random-effects meta-analysis were consistently more conservative than those from fixed-effects meta-analysis for all traits (Supplementary Figure 36, available as Supplementary data at *IJE* online).

Similar to analyses between HIV infection and cardiometabolic traits, we found marked heterogeneity among SMDs for all traits (Figure 4). However, stratified and meta-regression analyses did not consistently explain heterogeneity in estimates among studies (Table 2 and Supplementary Figures 37–44, available as Supplementary data at *IJE* online). Again, assessment of potential study-level effect-modification factors through meta-regression did not show clear evidence to suggest that these explained heterogeneity among studies (Table 2 and Supplementary Figures 37–44, available as Supplementary data at *IJE* online).

Based on visual assessments of forest and Galbraith plots, we found evidence of several outlying studies assessing the association between ART use and cardiometabolic traits (Supplementary Figures 28–35 and 45–52, available as Supplementary data at *IJE* online). Nevertheless, none of the combined SMDs that were associated with ART use materially changed during sequential exclusion (Table 3). We did, however, observe a change in SMD estimates for DBP on exclusion of one study, and for TGs on exclusion of one study, leading to associations between ART use and these cardiometabolic traits where there had previously been none. Individual combined SMDs for each sensitivity analysis are presented in Supplementary Tables 12–19, available as Supplementary data at *IJE* online.

### Individual participant data analysis

**Figure 5:**
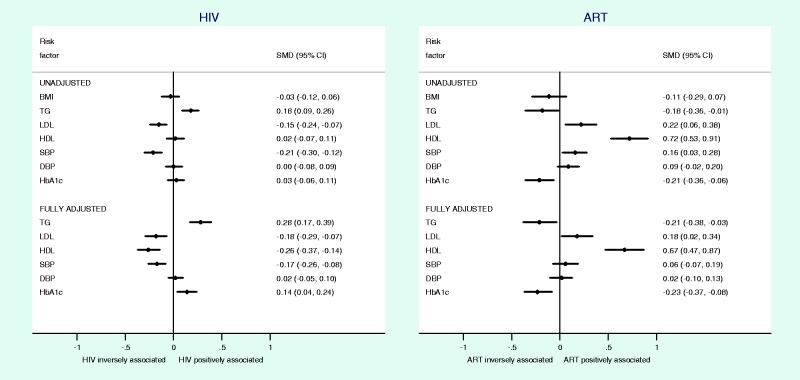
Participant-level data on the associations of HIV and ART with cardiometabolic traits in the General Population Cohort,^54^ adjusted for different amounts of individual-level confounding. Full adjustment includes adjustment for data clustering, ART exposure (when comparing HIV+ and HIV− subgroups), age, sex, BMI, education level and smoking status. SMD, standardized mean difference; CI, confidence interval; BMI, body mass index; TGs, triglycerides; LDL, low-density lipoprotein cholesterol; HDL, high-density lipoprotein cholesterol; SBP, systolic blood pressure; DBP, diastolic blood pressure; HbA1c, glycated haemoglobin; ART, antiretroviral therapy

To explore the potential effects of confounding on association estimates, we carried out individual participant data analysis in a subset of data adjusting for all potential confounders. Analysis of individual participant data from 5586 individuals in the GPC study, Uganda, was broadly consistent with summary estimates from meta-analysis for associations between HIV, ART and cardiometabolic traits. HIV infection was associated with higher TGs (SMD, 0.28; 95% CI, 0.17 to 0.39) and lower LDL (SMD, −0.18, 95% CI, −0.29 to −0.07), HDL (SMD, −0.26; 95% CI, −0.37 to −0.14) and SBP (SMD, −0.17; 95% CI, −0.26 to −0.08) when adjusted for age, sex, BMI, ART exposure, education level and smoking status and clustered by village and household status (Figure 5). In addition, we found a weak association between HIV infection and higher HbA1c levels (SMD, 0.14; 95% CI, 0.04 to 0.24) in the fully adjusted model (Figure 5). Comparing ART exposed and unexposed HIV-infected individuals, we found associations between ART use and higher LDL (SMD, 0.18; 95% CI, 0.02 to 0.34), HDL cholesterol levels (SMD, 0.67; 95% CI, 0.47 to 0.87), lower TGs (SMD, −0.21; 95% CI, −0.38 to −0.03) and HbA1c levels (SMD, −0.23; 95% CI, −0.37 to −0.08). In both analyses, fully adjusted estimates showed stronger associations than unadjusted estimates, suggesting that in this situation unadjusted estimates are more conservative than fully adjusted estimates. Sub-analyses comparing associations across all three subgroups (HIV−, HIV+/ART− and HIV+/ART+) in the GPC population are presented in Supplementary Table 3, available as Supplementary data at *IJE* online.

## Discussion

In this meta-analysis of data from up to 29 755 individuals in SSA, HIV infection was found to be associated with lower BMI, lower SBP, lower DBP, higher TGs and lower HDL levels. Among HIV-infected individuals, ART treatment was associated with higher LDL and HDL, as well as lower HbA1c levels. Heterogeneity among study estimates did not appear to be consistently explained by study-level factors, including potential confounders. These findings are broadly consistent with published results from populations of European descent.^78^^,^^79^ In a region with approximately 22.9 million cases of HIV and many millions of people on ART,^1^ it will be important to clarify these findings to reliably assess the need for monitoring and managing cardiometabolic risk in SSA populations.

Whereas several studies have documented lipid and glucose abnormalities in HIV-infected individuals and those treated with ART, the pathophysiology of these differences remains unclear. Higher levels of TGs in HIV-infected individuals have been attributed to higher concentrations of very-low-density lipoprotein cholesterol (VLDL) in plasma, and enrichment of LDL and HDL particles for TGs.^79^ TG clearance has been shown to be decreased in AIDS and HIV+ individuals, and elevated cytokine levels, such as IFN-alpha, might be involved in slowed clearance.^80^ It has been suggested that these changes may be due, in part, to the inflammatory effects of the viral infection.^79^ Several mechanisms have been outlined for the association between ART and dyslipidaemia, including reduced synthesis of cis-9-retinoic acid, leading to dysregulation of adipocyte differentiation and apoptosis, increased hepatic TG synthesis,^81^ increase in dense LDL particles, a shift towards TG-rich VLDL and increase in apolipoprotein C-III- and apolipoprotein E-containing particles. However, mechanisms are thought to be different for the various classes of ART drugs.^79^

Associations between HIV, ART and blood lipids observed in this meta-analysis are consistent with studies from Europe and North America, which show that HIV infection in ART-naive individuals is associated with hypertriglyceridaemia and lower HDL and LDL levels^78^^,^^79^ whereas ART use is associated with higher HDL and LDL levels.^78^^,^^82–84^ Both the magnitude and the direction of HIV and ART associations with HDL and LDL are consistent with reported estimates. We did not find an association between ART and TGs in this study, which is inconsistent with a meta-analysis of randomized clinical trials reporting a positive association between first-line ART and TGs, with stronger associations observed in protease inhibitor-treated patients.^82^ Furthermore, results for the association between ART exposure and TG were inconsistent between meta-analysis of summary data and individual participant data from the GPC. These inconsistencies are likely to be due to different treatment regimens across studies and infrequent use of protease inhibitors in comparison with nucleoside reverse transcriptase inhibitors and non-nucleoside reverse transcriptase inhibitors (NNRTI) in SSA.^85–87^ Indeed, regimens based on nevirapine (an NNRTI drug) are the most commonly used in the GPC HIV+ patient population, which may explain the inverse association between ART exposure and TG in the individual-level analysis, as previously noted.^87^

Similarly, the inverse association between HIV infection and BMI in SSA populations is consistent with previously published findings in populations of European descent.^88^ Advanced stages of HIV have been consistently associated with a rapid decrease in BMI.^89^ There is also clear evidence supporting the role of HIV infection and ART use in the pathogenesis of lipodystrophy, and the effects these changes in body-fat redistribution may have on cardiometabolic traits.^51^^,^^79–90^ However, in our individual-level dataset neither HIV nor ART is associated with differences in BMI. Thus, it is unclear what effect BMI has on the relationship among HIV, ART and cardiometabolic traits in these populations.

Our analyses found that individuals infected with HIV in SSA had lower DBP and SBP than uninfected controls, regardless of ART status. Previous studies assessing the associations between HIV, ART and blood pressure have been inconsistent, with some studies suggesting increased risk of hypertension with ART,^91^ some reporting no association with HIV or ART^92^^,^^93^ and others supporting the findings of this meta-analysis.^94^ There is no clear biological mechanism that might account for such associations. One explanation for our findings may be residual confounding in our meta-analysis of study-level data. Indeed, both BMI and blood pressure were inversely associated with HIV in our data. However, individual participant analysis in a subset of data showed the association between SBP and HIV infection was robust to adjustment for potential confounders. Nevertheless, we cannot exclude residual or unknown confounding as a possible explanation. Equally, although the lack of association between ART and blood pressure seen in our analysis supports previously published results,^92^^,^^95^only a small number of studies reported blood pressure measurements in both ART+ and ART- populations, suggesting these results require further evaluation.

We did not find an association between HIV infection, ART use and fasting blood glucose levels. Whereas this finding is consistent with findings from several large prospective^11^^,^^96^^,^^97^ and cross-sectional studies,^98^ it does not agree with findings from some large studies in populations of European descent that have reported associations between HIV infection, ART use and increased risk of T2D.^10–12^^,^^99^ One explanation for this may be the relative scarcity of relevant studies and the relatively small sample sizes in our data. Furthermore, the direction and magnitude of the association may differ in African populations, as suggested previously in analyses among African-American women in the Women’s Interagency HIV study.^100^ Although adjusted estimates from individual participant analyses suggested inverse associations between HIV and HbA1c, and positive associations between ART exposure and HbA1c, it must be noted that haemoglobin levels and red cell turnover may be altered by HIV infection and ART exposure, and HbA1c in these individuals may not be accurately representative of glycaemic status.^101^ Specific, large-scale prospective studies in sub-Saharan Africa are needed to more reliably assess these associations.

Differences in cardiometabolic traits among HIV−, HIV+ and ART users and non-users may have important implications for the management of people infected with HIV. Antiretroviral therapy has greatly improved the survival of HIV-infected patients living today; however, the mortality rates in HIV patients are still higher than in the general population and the proportion of deaths due to non-HIV-related causes including cardiometabolic diseases, is increasing.^102^^,^^103^ Dyslipidaemia is common among patients with HIV and has been shown to be associated with increased cardiovascular disease risk in this patient population.^80^^,^^104^ Furthermore, there is evidence to support an independent role of HIV infection on cardiometabolic disease risk, after accounting for traditional risk factors and exposure to ART.^79^ In the Data Collection on Adverse Events of Anti-HIV Drugs (DAD) cohort of 23 468 HIV-infected patients, higher total serum cholesterol and TGs and presence of diabetes were associated with an increased risk of myocardial infarction.^14^ Differences in average levels of cardiometabolic traits among subpopulations might also result in important differences in cardiovascular disease risk. For example, a 1-SD increase in LDL and HDL each were associated with a relative risk of 1.4 and 0.6, respectively, for coronary heart disease in the Atherosclerosis Risk in Communities Study (ARIC) examining 12 336 individuals, indicating that a change of 0.6 SD in HDL and/or 0.4 SD in LDL observed in our meta-analysis may have important implications for modifying cardiovascular risk in these groups.^105^

Findings from this meta-analysis should be interpreted within the context of its strengths and limitations. One of the strengths of the study is the large sample of individuals examined from different studies. We used a comprehensive and systematic search strategy examining two separate journal databases and contacted authors of studies for information on unpublished data and grey literature. Although the restriction of our search to only PubMed and EMBASE could be seen as a limitation, we feel that the combination of cited reference list searches and direct author contact helps ameliorate this concern. Furthermore, study eligibility was rigorously assessed by two independent reviewers, making assessment bias unlikely. However, we acknowledge that restricting this systematic review to English language articles may not be representative of the non-English literature. We were unable to correct for confounders at the individual level for all studies. Although none of the study-level characteristics and indices for study design substantially explained the heterogeneity among estimates from studies, we cannot rule out confounding at the individual level. Meta-regression approaches also have limited statistical power with sparse data. However, we were able to evaluate the potential effects of residual confounding with individual participant data analysis in a large cohort study, comprising nearly one-fifth of the overall data. Individual-level adjustment for confounders in a subset of data showed that SMDs are likely to be under-estimated (more conservative) in unadjusted analysis across most traits, suggesting that association estimates from summary data are unlikely to be overestimated. Unexplained heterogeneity could be attributed to one or more of several factors, including differences in study design and objective, differential confounding in each study due to age, sex, participant CD4 count or WHO stage, type and duration of ART treatment, co-infections or differences in data collection and laboratory assays. However, our findings are broadly consistent with published findings in populations of European descent,^78^^,^^79^^,^^106^ as well as studies using individual-level data to assess these associations.^35^^,^^57^^,^^107^

An additional limitation of this study is the inability to delineate associations by ART drug class, due to insufficient data. Such analyses would be invaluable in understanding these associations, and their results would likely be of direct clinical relevance. Nevertheless, despite a lack of specific information on drug class, we identified associations between general ART use and differences in several cardiometabolic traits. It is likely that combining the impact of several different drugs in a single analysis would underestimate the individual effects of each drug.

Based on data presented here, the cardiometabolic consequences of HIV infection and ART exposure in SSA may be important. With a rapid increase in ART use over the past decade,^1^ an increasing number of SSA individuals are receiving treatment.^3^ As people live longer with HIV, it will become increasingly important to monitor their risk of other diseases. The HIV Medicine Association of the Infectious Disease Society of America, and the Adult AIDS Clinical Trials Group, published guidelines specifically for management of dyslipidaemia in HIV-infected individuals in 2003.^15^ Following this, the European AIDS Clinical Society (EACS) also published guidelines on the prevention and management of metabolic disease in HIV infection in 2008.^108^ Both these sets of guidelines have been based largely on evidence from studies in European populations and the impact of HIV infection and ART use on metabolic traits, and the utility of early screening and treatment in populations from SSA remains largely unknown. There is evidence to suggest that baseline metabolic profiles^20^ and associations between HIV and ART and metabolic risk factors may be different in different ethnic populations,^109^ with HIV-infected African-Americans being at higher risk of acute MI in comparison with individuals of European descent.^18^^,^^19^^,^^109^^,^^110^ This emphasises the need to examine these factors in SSA, where the burden of HIV infection is the greatest. Our results suggest that, with further evaluation, there may be a need to monitor cardiometabolic traits in HIV-infected individuals in SSA. One mechanism to achieve this, in the context of resource-poor settings, is to integrate care of chronic HIV with that of cardiometabolic diseases.^111^ Such routine monitoring has the potential to improve the management of cardiovascular disease among HIV-infected and ART-exposed individuals.^111^

The results of this meta-analysis suggest that HIV infection and ART treatment are both associated with differences in cardiometabolic traits compared with HIV-uninfected or ART-naïve patients in SSA. Individual-level associations from a subset of 5586 individuals, adjusted for several major cardiometabolic confounders, were generally consistent with study-level summary results, suggesting that the results from meta-analysis are likely to be robust to major confounding. To our knowledge, this is the first comprehensive study examining the association between HIV and cardiometabolic traits by a meta-analysis of published and unpublished data from SSA. These findings may have important implications for management of HIV in SSA, given the increasing use of ART and improved life expectancy among HIV-infected individuals in this region, and could provide a framework for further research aimed towards the development of specific guidelines for assessment and management of cardiometabolic risk in HIV-infected individuals in the region. More comprehensive analyses, including the collection of prospective observational data, and a pooled analysis of individual-level cross-sectional data from the region are needed to clarify these findings and reliably assess the need for monitoring and managing cardiometabolic risk in populations in SSA.

## Supplementary Data

Supplementary data are available at *IJE* online.

Supplementary Data
