# Association of HIV and ART with cardiometabolic traits in sub-Saharan Africa: a systematic review and meta-analysis

**DOI:** 10.1093/ije/dyw312

**Published:** 2016-11-18

**Authors:** D.G. Dillon

*Int J Epidemiol* 2013;42:1754-1771, doi:10.1093/ije/dyt198

During the preparation of this article, owing to an administrative oversight between the collaborating institutions, we omitted Dr Dermot Maher from the author list. Dr Maher was involved in the early phases of the General Population Cohort (GPC) Study, data from which were used in the individual-level participant data analyses in this manuscript. His role included contributing to the GPC study protocol development, management of initial research ethics application for the GPC, and organisation of initial field programmes at MRC/UVRI Uganda Research Unit on AIDS.

As a result, we request that Dr Maher is included in the author list. This results in the following correction:

David G Dillon,^1,2^ Deepti Gurdasani,^1,2^ Johanna Riha,^1,2^ Kenneth Ekoru,^1,2,3^ Gershim Asiki,^3^ Billy N Mayanja,^3^**Dermot Maher,^3^** Naomi S Levitt,^4^ Nigel J Crowther,^5^ Moffat Nyirenda,^6^ Marina Njelekela,^7^ Kaushik Ramaiya,^8^ Ousman Nyan,^9^ Olanisun O Adewole,^10^ Kathryn Anastos,^11^ Livio Azzoni,^12^ W Henry Boom,^13^ Caterina Compostella,^14^ Joel A Dave,^15^ Halima Dawood,^16^ Christian Erikstrup,^17^ Carla M Fourie,^18^ Henrik Friis,^19^ Annamarie Kruger,^20^ John A Idoko,^21^ Chris T Longenecker,^22^ Suzanne Mbondi,^23^ Japheth E Mukaya,^24^ Eugene Mutimura,^11^ Chiratidzo E Ndhlovu,^25^ George Praygod,^26^ Eric W Pefura Yone,^27^ Mar Pujades-Rodriguez,^28,29^ Nyagosya Range,^26^ Mahmoud U Sani,^30^ Aletta E Schutte,^18^ Karen Sliwa,^31^ Phyllis C Tien,^32^ Este H Vorster,^33^ Corinna Walsh,^34^ Rutendo Zinyama,^35^ Fredirick Mashili,^7^ Eugene Sobngwi,^36,37^ Clement Adebamowo,^38,39^ Anatoli Kamali,^3^ Janet Seeley,^3^ Elizabeth H Young,^1,2^ Liam Smeeth,^40^ Ayesha A Motala,^41^ Pontiano Kaleebu,^3^ Manjinder S Sandhu^1,2^* and on behalf of the African Partnership for Chronic Disease Research (APCDR).

^1^Department of Public Health and Primary Care, Institute of Public Health, University of Cambridge, Cambridge, UK,

^2^Genetic Epidemiology Group, Wellcome Trust Sanger Institute, Hinxton, Cambridge, UK,

^3^MRC/UVRI Uganda Research Unit on AIDS, Entebbe, Uganda,

^4^Division of Diabetic Medicine and Endocrinology, Department of Medicine, University of Cape Town, Cape Town, South Africa; Chronic Diseases Initiative in Africa,

^5^Department of Chemical Pathology, National Health Laboratory Service, University of the Witwatersrand Medical School, Johannesburg, South Africa,

^6^Malawi-Liverpool-Wellcome Trust Clinical Research Programme, Blantyre, Malawi,

^7^Department of Physiology, Muhimbili University of Health and Allied Sciences, Dar es Salaam, Tanzania,

^8^Department of Medicine, Muhimbili University of Health and Allied Sciences, Dar es Salaam, Tanzania,

^9^Royal Victoria Teaching Hospital, School of Medicine, University of The Gambia, Banjul, The Gambia,

^10^Department of Medicine, Obafemi Awolowo University, Ile Ife, Nigeria,

^11^Women’s Equity in Access to Care &Treatment, Kigali, Rwanda,

^12^HIV-1 Immunopathogenesis Laboratory, Wistar Institute, Philadelphia, PA,

^13^Tuberculosis Research Unit, Department of Medicine, Case Western Reserve University, Cleveland, OH,

^14^Department of Medical and Surgical Sciences, University of Padua, Padua, Italy,

^15^Division of Diabetic Medicine and Endocrinology, Department of Medicine, University of Cape Town, Cape Town, South Africa,

^16^Infectious Diseases Unit, Department of Medicine, Grey’s Hospital, Pietermaritzburg, South Africa,

^17^Department of Clinical Immunology, Aarhus University Hospital, Aarhus, Denmark,

^18^HART (Hypertension in Africa Research Team), North-West University, Potchefstroom, South Africa,

^19^Department of Nutrition, Exercise and Sports, Faculty of Science, University of Copenhagen, Copenhagen, Denmark,

^20^Africa Unit for Transdisciplinary Health Research (AUTHeR), North-West University, Potchefstroom, South Africa,

^21^Department of Medicine, Jos University Teaching Hospital, Jos, Nigeria,

^22^University Hospitals Case Medical Center, Cleveland, OH,

^23^German Development Cooperation (GTZ), Yaounde, Cameroon,

^24^Department of Medicine, Makerere University, Kampala, Uganda,

^25^Clinical Epidemiology Resource Training Centre, University of Zimbabwe College of Health Sciences, Harare, Zimbabwe,

^26^National Institute for Medical Research, Dar es Salaam, Tanzania,

^27^Chest Unit of Jamot Hospital, Yaounde, Cameroon,

^28^Epicentre, Médecins Sans Frontie`res, Paris, France,

^29^Clinical Epidemiology Group, Department of Epidemiology and Public Health, University College London, London, UK,

^30^Cardiology Unit, Department of Medicine, Aminu Kano Teaching Hospital, Kano, Nigeria,

^31^Soweto Cardiovascular Research Unit, Chris Hani Baragwanath Hospital, University of the Witwatersrand, Johannesburg, South Africa,

^32^Department of Medicine, University of California, San Francisco, CA, 33Faculty of Health Sciences, North-West University, Potchefstroom, South Africa,

^34^Department of Nutrition and Dietetics, University of the Free State, Bloemfontein, South Africa,

^35^Medical Research Council of Zimbabwe, Department of Medical Laboratory Sciences, University of Zimbabwe, Harare, Zimbabwe,

^36^Faculty of Medicine and Biomedical Sciences, University of Yaounde 1, Yaounde, Cameroon,

^37^Institute of Health and Society, University of Newcastle, Newcastle, UK,

^38^Institute of Human Virology, Abuja, Nigeria,

^39^Department of Epidemiology and Public Health, Institute of Human Virology and Greenebaum Cancer Center, University of Maryland School of Medicine, Baltimore, MD,

^40^Faculty of Epidemiology and Population Health, London School of Hygiene and Tropical Medicine, London, UK

^41^Department of Diabetes and Endocrinology, Nelson R. Mandela School of Medicine, University of KwaZulu-Natal, Durban, South Africa

*Corresponding author. International Health Research Group, Department of Public Health and Primary Care, University of Cambridge, Cambridge, UK. E-mail: ms23@sanger.ac.uk

We apologise for this oversight.

