# Supplementary material for: Association of HIV and ART with cardiometabolic traits in sub-Saharan Africa: a systematic review and meta-analysis
Source: Int J Epidemiol. 2014 Jan 8;42(6):1754–71. doi: 10.1093/ije/dyt198 (PMC3887568; doi:10.1093/ije/dyt198)
Supplement: Supplementary Data [file supp_42_6_1754__index.html]

Supplementary Data 

# Association of HIV and ART with cardiometabolic traits in sub-Saharan Africa: a systematic review and meta-analysis

## Supplementary Data

files

**Files in this Data Supplement:**

- Supplementary Data - docx file
- Supplementary Data - xlsx file
- Supplementary Data - xlsx file
