# Supplementary material for: Association of HIV and ART with cardiometabolic traits in sub-Saharan Africa: a systematic review and meta-analysis
Source: Int J Epidemiol. 2014 Jan 8;42(6):1754–71. doi: 10.1093/ije/dyt198 (PMC3887568; doi:10.1093/ije/dyt198)
Supplement: Supplementary Data [file supp_dyt198_ije-2013-03-0317-File002.docx]

**Associations among HIV, ART and cardiometabolic traits in sub-Saharan Africa: a systematic review and meta-analysis**

**Supplementary tables and figures**

**David G. Dillon, Deepti Gurdasani, Johanna Riha, Kenneth Ekoru, Asiki Gershim, Billy N Mayanja, Naomi S. Levitt, Nigel J. Crowther, Moffat Nyirenda, Marina Njelekela, Kaushik Ramaiya, Ousman Nyan, Olanisun O. Adewole, Kathryn Anastos, Livio Azzoni, W. Henry Boom, Caterina Compostella, Joel A. Dave, Halima Dawood, Christian Erikstrup, Carla M. Fourie, Henrik Friis, Annamarie Kruger, John A. Idoko, Chris T. Longenecker, Suzanne Mbondi, Japheth E. Mukaya, Eugene Mutimura, Chiratidzo E. Ndhlovu, Chidzewere Nzou, George Praygod, Eric W. Pefura Yone, Mar Pujades-Rodriguez, Nyagosya Range, Mahmoud U. Sani, Muhammad Sanusi, Aletta E. Schutte, Karen Sliwa, Phyllis C. Tien, Este H. Vorster, Corinna Walsh, Rutendo Zinyama, Fredirick Mashili, Eugene Sobngwi, Clement Adebamowo, Anatoli Kamali, Janet Seeley, Elizabeth H. Young, Liam Smeeth, Ayesha A. Motala, Pontiano Kaleebu, Manjinder S. Sandhu on behalf of the African Partnership for Chronic Disease Research**

**Table of Contents**

| **Study identification and exclusion** |  |
| --- | --- |
| *Supplementary Figure 1:* PubMed search strategy | S3 |
| *Supplementary Figure 2:* EMBASE search strategy | S4 |
| *Supplementary Table 1:* Article list, exclusion status and reason | S5 |
|  |  |
| **Study level data summary** |  |
| *Supplementary Table 2:* Study level summary statistics from studies contributing to this meta-analysis | S6 |
|  |  |
| **Individual level data summary** |  |
| *Supplementary Table 3:* Descriptive and Cardiometabolic Characteristics by Exposure to HIV and ART in the General Population Cohort (MRC/UVRI – Unpublished) | S7 |
|  |  |
| **HIV associations with cardiometabolic traits** |  |
| *Supplementary Figures 3 – 10:* Meta-analyses of the associations between HIV infection and individual cardiometabolic traits | S8 – S15 |
| *Supplementary Figure 11:* Summary of overall estimates from fixed effects meta-analyses of HIV associations with individual cardiometabolic traits | S16 |
| *Supplementary Figures 12 – 19:* Stratified analyses of heterogeneity of the associations between HIV and individual cardiometabolic traits | S17 – S24 |
| *Supplementary Figures 20 – 27:* Galbraith plots of the associations between HIV and individual cardiometabolic traits | S25 – S32 |
| *Supplementary Tables 4 – 11:* Sensitivity analyses of the change in combined standardised mean difference estimates for the associations between HIV and individual cardiometabolic traits after sequential exclusion of single studies | S33 – S40 |
|  |  |
| **ART associations with cardiometabolic traits** |  |
| *Supplementary Figures 28 – 35:* Meta-analyses of the associations between ART use and individual cardiometabolic traits | S41 – S48 |
| *Supplementary Figure 36:* Summary of overall estimates from fixed effects meta-analyses of ART associations with individual cardiometabolic traits | S49 |
| *Supplementary Figures 37 – 44:* Stratified analyses of heterogeneity of the associations between ART and individual cardiometabolic traits | S50 – S57 |
| *Supplementary Figures 45 – 52:* Galbraith plots of the associations between ART and individual cardiometabolic traits | S58 – S65 |
| *Supplementary Tables 12 – 19:* Sensitivity analyses of the change in combined standardised mean difference estimates for the associations between ART and individual cardiometabolic traits after sequential exclusion of single studies | S66 – S73 |

***Supplementary Figure 1:* PubMed search strategy**

*Limits – Humans, English, Adult*

(“Africa”[MeSH Terms] OR “Africa South of the Sahara”[MeSH Terms] OR sub-Saharan Africa[All Fields] OR “African”[All Fields] OR “Africa”[All Fields] OR “Senegal”[All Fields] OR “Gambia”[All Fields] OR “Guinea Bissau”[All Fields] OR “Guinea”[All Fields] OR “Sierra Leone”[All Fields] OR “Liberia”[All Fields] OR “Ivory Coast”[All Fields] OR “Ghana”[All Fields] OR “Togo”[All Fields] OR “Benin”[All Fields] OR “Burkina Faso”[All Fields] OR “Nigeria”[All Fields] OR “Cameroon”[All Fields] OR “Central African Republic”[All Fields] OR “Eritrea”[All Fields] OR “Ethiopia”[All Fields] OR “Djibouti”[All Fields] OR “Somalia”[All Fields] OR “Kenya”[All Fields] OR “Uganda”[All Fields] OR “Democratic Republic of the Congo”[All Fields] OR “Sudan” [All Fields] OR “Congo”[All Fields] OR “Equatorial Guinea”[All Fields] OR “Gabon”[All Fields] OR “Angola”[All Fields] OR “Zambia”[All Fields] OR “Rwanda”[All Fields] OR “Burundi”[All Fields] OR “Tanzania”[All Fields] OR “Malawi”[All Fields] OR “Mozambique”[All Fields] OR “Madagascar”[All Fields] OR “Zimbabwe”[All Fields] OR “Botswana”[All Fields] OR “Namibia”[All Fields] OR “Swaziland”[All Fields] OR “Lesotho”[All Fields] OR “South Africa”[All Fields] OR “Sao Tome”[All Fields] OR “Principe”[All Fields])

AND

(("HIV"[MeSH Terms] OR "Acquired Immunodeficiency Syndrome"[All Fields] OR "hiv"[MeSH Terms] OR HIV[All Fields] OR “AIDS”[All Fields]) OR (“antiretroviral therapy, highly active”[MeSH Terms] OR “antiretroviral”[All Fields] OR “ART”[All Fields] OR “HAART”[All Fields] OR “anti-retroviral”[All Fields] OR "Nucleotide reverse transcriptase inhibitor"[All Fields] OR "NRTI"[All Fields] OR "Non-nucleoside reverse transcriptase inhibitor"[All Fields] OR "NNRTI"[All Fields] OR "Protease inhibitor"[All Fields] OR "PI"[All Fields] OR "PIs"[All Fields] OR “lopinavir”[All Fields] OR “ritonavir”[All Fields] OR “lamivudine”[All Fields] OR “zidovudine”[All Fields] OR “stavudine”[All Fields] “nevirapine”[All Fields] OR “efavirenz”[All Fields] OR "tenofovir" [All Fields] OR "emtricitabine" [All Fields] OR "atazanavir" [All Fields] OR "darunavir" [All Fields]))

AND

(("dyslipidemias”[MeSH Terms] OR “dyslipidemia”[All Fields] OR “hyperlipidemia”[All Fields] OR “hypercholesterolemia”[All Fields] OR “hypertriglyceridemia”[All Fields] OR “cholesterol”[All Fields] OR “triglyceride”[All Fields] OR “triglycerides”[All Fields] OR “HDL”[All Fields] OR “LDL”[All Fields] OR “VLDL”[All Fields] OR “Lp(a)”[All Fields] OR “hyperlipoproteinemia”[All Fields] OR “lipoprotein(a)”[All Fields] OR “hyperlipidaemia”[All Fields] OR “hypercholesterolaemia”[All Fields] OR “hypertriglyceridaemia”[All Fields]

OR

"Heart Diseases"[MeSH Terms] OR “cardiovascular disease”[All Fields] OR “heart disease”[All Fields] OR “CVD”[All Fields] OR “cardiovascular diseases”[MeSH Terms])

OR

("diabetes mellitus"[MeSH Terms] OR “diabetes”[All Fields] OR “hyperglycemia”[All Fields] OR “hyperglycaemia”[All Fields] OR “glucose”[All Fields] OR “IGT”[All Fields] OR “IFG”[All Fields] OR “HbA1c”[All Fields] OR “Hemoglobin A, Glycosylated”[MeSH Terms]

OR

"insulin resistance"[MeSH Terms] OR “insulin”[All Fields] OR “hyperinsulinemia”[All Fields] OR “hyperinsulinaemia”[All Fields])

OR

(“hypertension”[MeSH Terms] OR “hypertension”[All Fields] OR “Blood Pressure”[MeSH Terms] OR “blood pressure”[All Fields] OR “systolic blood pressure”[All Fields] OR “diastolic blood pressure”[All Fields] OR “SBP”[All Fields] OR “DBP”[All Fields])

OR

(“body mass index”[MeSH Terms] OR “body mass index”[All Fields] OR “BMI”[All Fields] OR “lipodystrophy”[All Fields] OR “lipodystrophy”[MeSH Terms] OR “HIV-Associated Lipodystrophy Syndrome”[MeSH Terms]))

***Supplementary Figure 2:* EMBASE search strategy**

*Limits – Humans, English, EMBASE, Adult*

1. (Africa or African or Senegal or Gambia or "Guinea Bissau" or Guinea or "Sierra Leone" or Liberia or "Ivory Coast" or Ghana or Togo or Benin or "Burkina Faso" or Nigeria or Cameroon or "Central African Republic" or Eritrea or Ethiopia or Djibouti or Somalia or Kenya or Uganda or "Democratic Republic of the Congo" or Congo or "Equitorial Guinea" or Gabon or Angola or Zambia or Rwanda or Burundi or Tanzania or Malawi or Mozambique or Madagascar or Zimbabwe or Namibia or Botswana or Swaziland or Lesotho or "South Africa" or “Sao Tome” or Principe).af.

2. (HIV or "Acquired Immunodeficiency Syndrome" or antiretroviral or ART or HAART or "anti-retroviral" or "Nucelotide reverse transcriptase inhibitor" or NRTI or "Non-nucleoside reverse transcriptase inhibitor" or NNRTI or "Protease inhibitor" or PI or PIs or lopinavir or ritonavir or lamivudine or zidovudine or stavudine or nevirapine or efavirenz or tenofovir or emtricitabine or atazanavir or darunavir).af.

3. (dyslipidemias or dyslipidemia or hyperlipidemia or hypercholesterolemia or hypertriglyceridemia or cholesterol or triglyceride or triglycerides or HDL or LDL or VLDL or Lp a or hyperlipoproteinemia or lipoprotein a or hyperlipidaemia or hypercholesterolaemia or hypertriglyceridaemia or "heart disease" or "cardiovascular disease" or CVD or "heart diseases" or diabetes or hyperglycemia or hyperglycaemia or glucose or IGT or IFG or HbA1c or "Hemoglobin A, Glycosylated" or "insulin resistance" or insulin or hyperinsulinemia or hyperinsulinaemia or hypertension or "blood pressure" or SBP or DBP or "systolic blood pressure" or "diastolic blood pressure" or "body mass index" or BMI or Lipodystrophy or "HIV-associated lipodystrophy syndrome").af.

4. 1 and 2 and 3

***Supplementary Table 1:* Article list, exclusion status and reason**

Please see attached file – Supplementary Table 1

***Supplementary Table 2:* Study level summary statistics from studies contributing to this meta-analysis**

Please see attached file – Supplementary Table 2

***Supplementary Table 3:* Descriptive and Cardiometabolic Characteristics by Exposure to HIV and ART in the General Population Cohort (MRC/UVRI – Unpublished)**

|  | **Total population** | **HIV-** | **HIV+/ART-** | **HIV+/ART+** |
| --- | --- | --- | --- | --- |
|  | **n (%)** | **n (%)** | **n (%)** | **n (%)** |
| **Number of individuals** | 5,586 | 5,028 | 337 | 221 |
|  |  |  |  |  |
| **Descriptive characteristics** |  |  |  |  |
| Sex |  |  |  |  |
| Male | 2,272 (40.7%) | 2,084 (41.5%) | 118 (35.0%) | 70 (31.7%) |
| Female | 3,314 (59.3%) | 2,944 (58.6%) | 219 (65.0%) | 151 (68.3%) |
| Age |  |  |  |  |
| 18-24 | 1,149 (20.6%) | 1,095 (21.8%) | 44 (13.1%) | 10 (4.5%) |
| 25-44 | 2,426 (43.4%) | 2,092 (41.6%) | 215 (63.8%) | 119 (53.9%) |
| ≥45 | 2,011 (36.0%) | 1,841 (36.6%) | 78 (21.2%) | 92 (41.6%) |
| Education level |  |  |  |  |
| None | 690 (12.4%) | 624 (12.4%) | 40 (11.9%) | 26 (11.8%) |
| Some Primary | 2,162 (38.7%) | 1,904 (37.9%) | 154 (45.7%) | 104 (47.1%) |
| Some secondary | 1,137 (20.4%) | 1,022 (20.3%) | 71 (21.1%) | 44 (19.9%) |
| Above secondary | 1,597 (28.6%) | 1,478 (29.4%) | 72 (21.4%) | 47 (21.3%) |
| Regular smoking |  |  |  |  |
| Never | 4,779 (86.4) | 4,326 (86.9%) | 269 (80.8%) | 184 (84.4%) |
| Previous | 476 (8.6%) | 414 (8.3%) | 46 (13.8%) | 16 (7.3%) |
| Current | 276 (5.0%) | 240 (4.8%) | 18 (5.4%) | 18 (8.3%) |
| Type of ART regimen |  |  |  |  |
| AZT+3TC+TDF | - - | - - | - - | 1 (0.5%) |
| 3TC+TDF+NVP | - - | - - | - - | 5 (2.3%) |
| 3TC+TDF+LPV/r | - - | - - | - - | 6 (2.7%) |
| AZT+3TC+EFV | - - | - - | - - | 11 (5.0%) |
| 3TC+TDF+EFV | - - | - - | - - | 15 (6.8%) |
| AZT+3TC+NVP | - - | - - | - - | 183 (82.8%) |
|  |  |  |  |  |
| **Cardiometabolic risk profile** |  |  |  |  |
| Raised blood pressure | 1,108 (19.8%) | 1,023 (20.4%) | 45 (13.4%) | 40 (18.1%) |
| High cholesterol | 426 (7.7%) | 389 (7.8%) | 16 (4.8%) | 21 (9.6%) |
| High HbA1c | 13 (0.2%) | 13 (0.3%) | 0 (0.0%) | 0 (0.0%) |
| Raised triglycerides | 293 (5.3%) | 258 (5.1%) | 23 (6.8%) | 12 (5.5%) |
| Overweight | 843 (15.8%) | 768 (16.0%) | 49 (15.3%) | 26 (12.3%) |
|  |  |  |  |  |
| **Cardiometabolic traits** | n | mean (SD) | mean (SD) | mean (SD) |
| TGs | 5,571 | 1.19 (0.62) | 1.33 (0.74) *** | 1.27 (0.86) |
| Total cholesterol | 5,569 | 3.67 (1.04) | 3.47 (0.90) *** | 3.92 1.07) *** § |
| HDL | 5,567 | 1.05 (0.41) | 0.94 (0.43) *** | 1.24 (0.52) *** § |
| LDL | 5,574 | 2.10 (0.81) | 1.91 (0.72) *** | 2.08 (0.75) ‡ |
| SBP | 5,584 | 124.7 (21.4) | 118.9 (14.7) *** | 122.2 (16.8) † |
| DBP | 5,584 | 75.5 (15.6) | 75.0 (9.7) | 76.4 (10.0) |
| BMI | 5,325 | 22.0 (3.8) | 22.0 (3.5) | 21.6 (4.5) |
| Waist/hip ratio | 5,343 | 0.84 (0.06) | 0.85 (0.05) | 0.85 (0.05) * |
| HbA1c | 5,551 | 3.37 (0.68) | 3.44 (0.63) | 3.30 (0.56) ‡ |

* *P* value ≤ 0.05 when compared to HIV- population; ** *P* value ≤ 0.01 when compared to HIV- population; *** *P* value ≤ 0.001 when compared to HIV- population; † *P* value ≤ 0.05 when compared to HIV+/ART- population; ‡ *P* value ≤ 0.01 when compared to HIV+/ART- population; § *P* value ≤ 0.001 when compared to HIV+/ART- population; SD=Standard deviation; TGs=Triglycerides; HDL=High density lipoprotein cholesterol; LDL=Low density lipoprotein cholesterol; BMI=Body mass index; SBP=Systolic blood pressure; DBP=Diastolic blood pressure; HbA1c=Glycated hemoglobin; Raised blood pressure defined as SBP ≥ 140 mmHg, DBP ≥ 90 mmHg or reported treatment for raised blood pressure, high cholesterol defined as total cholesterol > 5.1 mmol/L, high HbA1c as HbA1c ≥ 6.5%, raised triglycerides as triglycerides > 2.3 mmol/L, and overweight as BMI ≥ 25 kg/m^2^; Smoking status derived from self-reported information; AZT=Zidovudine; 3TC= Lamivudine; NVP=Nevirapine; EFV=Efavirenz; LVP/r=Lopinovir/ ritonavir; TDF=Tenofovir; - - =Not applicable

*Supplementary Figure 3:* Meta-analysis of the association between HIV infection and BMI

SMD=Standardised mean difference; CI=Confidence interval; SD=Standard deviation; BMI=Body mass index

*Supplementary Figure 4:* Meta-analysis of the association between HIV infection and TGs

SMD=Standardised mean difference; CI=Confidence interval; SD=Standard deviation; TGs=Triglycerides

*Supplementary Figure 5:* Meta-analysis of the association between HIV infection and LDL

SMD=Standardised mean difference; CI=Confidence interval; SD=Standard deviation; LDL=Low density lipoprotein cholesterol

*Supplementary Figure 6:* Meta-analysis of the association between HIV infection and HDL

SMD=Standardised mean difference; CI=Confidence interval; SD=Standard deviation; HDL=High density lipoprotein cholesterol

*Supplementary Figure 7:* Meta-analysis of the association between HIV infection and SBP

SMD=Standardised mean difference; CI=Confidence interval; SD=Standard deviation; SBP=Systolic blood pressure

*Supplementary Figure 8:* Meta-analysis of the association between HIV infection and DBP

SMD=Standardised mean difference; CI=Confidence interval; SD=Standard deviation; DBP=Diastolic blood pressure

*Supplementary Figure 9:* Meta-analysis of the association between HIV infection and glucose

SMD=Standardised mean difference; CI=Confidence interval; SD=Standard deviation

*Supplementary Figure 10:* Meta-analysis of the association between HIV infection and HbA1c

SMD=Standardised mean difference; CI=Confidence interval; SD=Standard deviation; HbA1c=Glycated hemoglobin

*Supplementary Figure 11:* Summary of overall estimates from fixed effects meta-analyses of HIV associations with individual cardiometabolic traits

SMD=Standardised mean difference; CI=Confidence interval; BMI=Body mass index; TGs=Triglycerides; LDL=Low density lipoprotein cholesterol; HDL=High density lipoprotein cholesterol; SBP=Systolic blood pressure; DBP=Diastolic blood pressure; HbA1c=Glycated hemoglobin

*Supplementary Figure 12:* Stratified analysis of heterogeneity of the association between HIV and BMI

SMD=Standardised mean difference; CI=Confidence interval; BMI=Body mass index

*Supplementary Figure 13:* Stratified analysis of heterogeneity of the association between HIV and TGs

SMD=Standardised mean difference; CI=Confidence interval; TGs=Triglycerides

*Supplementary Figure 14:* Stratified analysis of heterogeneity of the association between HIV and LDL

SMD=Standardised mean difference; CI=Confidence interval; LDL=Low density lipoprotein cholesterol

*Supplementary Figure 15:* Stratified analysis of heterogeneity of the association between HIV and HDL

SMD=Standardised mean difference; CI=Confidence interval; HDL=High density lipoprotein cholesterol

*Supplementary Figure 16:* Stratified analysis of heterogeneity of the association between HIV and SBP

SMD=Standardised mean difference; CI=Confidence interval; SBP=Systolic blood pressure

*Supplementary Figure 17:* Stratified analysis of heterogeneity of the association between HIV and DBP

SMD=Standardised mean difference; CI=Confidence interval; DBP=Diastolic blood pressure

*Supplementary Figure 18:* Stratified analysis of heterogeneity of the association between HIV and glucose

SMD=Standardised mean difference; CI=Confidence interval

*Supplementary Figure 19:* Stratified analysis of heterogeneity of the association between HIV and HbA1c

SMD=Standardised mean difference; CI=Confidence interval; HbA1c=Glycated hemoglobin

*Supplementary Figure 20:* Galbraith plot of the association between HIV and BMI

Dots represent studies. Unweighted regression line with 95% confidence intervals superimposed as three parallels lines. In the absence of heterogeneity, we would expect all studies to lie within the 95% confidence intervals; b=Standardised mean difference estimate; se=Standard error for each estimate (b); BMI=Body mass index

*Supplementary Figure 21:* Galbraith plot of the association between HIV and TGs

Dots represent studies. Unweighted regression line with 95% confidence intervals superimposed as three parallels lines. In the absence of heterogeneity, we would expect all studies to lie within the 95% confidence intervals; b=Standardised mean difference estimate; se=Standard error for each estimate (b); TGs=Triglycerides

*Supplementary Figure 22:* Galbraith plot of the association between HIV and LDL

Dots represent studies. Unweighted regression line with 95% confidence intervals superimposed as three parallels lines. In the absence of heterogeneity, we would expect all studies to lie within the 95% confidence intervals; b=Standardised mean difference estimate; se=Standard error for each estimate (b); LDL=Low density lipoprotein cholesterol

*Supplementary Figure 23:* Galbraith plot of the association between HIV and HDL

Dots represent studies. Unweighted regression line with 95% confidence intervals superimposed as three parallels lines. In the absence of heterogeneity, we would expect all studies to lie within the 95% confidence intervals; b=Standardised mean difference estimate; se=Standard error for each estimate (b); HDL=High density lipoprotein cholesterol

*Supplementary Figure 24:* Galbraith plot of the association between HIV and SBP

Dots represent studies. Unweighted regression line with 95% confidence intervals superimposed as three parallels lines. In the absence of heterogeneity, we would expect all studies to lie within the 95% confidence intervals; b=Standardised mean difference estimate; se=Standard error for each estimate (b); SBP=Systolic blood pressure

*Supplementary Figure 25:* Galbraith plot of the association between HIV and DBP

Dots represent studies. Unweighted regression line with 95% confidence intervals superimposed as three parallels lines. In the absence of heterogeneity, we would expect all studies to lie within the 95% confidence intervals; b=Standardised mean difference estimate; se=Standard error for each estimate (b); DBP=Diastolic blood pressure

*Supplementary Figure 26:* Galbraith plot of the association between HIV and glucose

Dots represent studies. Unweighted regression line with 95% confidence intervals superimposed as three parallels lines. In the absence of heterogeneity, we would expect all studies to lie within the 95% confidence intervals; b=Standardised mean difference estimate; se=Standard error for each estimate (b)

*Supplementary Figure 27:* Galbraith plot of the association between HIV and HbA1c

Dots represent studies. Unweighted regression line with 95% confidence intervals superimposed as three parallels lines. In the absence of heterogeneity, we would expect all studies to lie within the 95% confidence intervals; b=Standardised mean difference estimate; se=Standard error for each estimate (b); HbA1c=Glycated hemoglobin

*Supplementary Table 4:* Sensitivity analysis of the change in combined standardised mean difference estimates for the association between HIV and BMI after sequential exclusion of single studies

| Excluded study | Combined SMD (95% CI) |
| --- | --- |
| - - | -0.32 (-0.45 to -0.18) |
| Addo – 2011 | -0.30 (-0.44 to -0.17) |
| Adewole – 2010 | -0.26 (-0.39 to -0.14) |
| Becker – 2011 | -0.31 (-0.45 to -0.18) |
| Ceffa – 2007 | -0.32 (-0.46 to -0.18) |
| Compostella – 2008 | -0.31 (-0.44 to -0.18) |
| Erkistrup – 2007 | -0.31 (-0.45 to -0.18) |
| Ezechi – 2010 | -0.29 (-0.41 to -0.18) |
| Friis – 2002 | -0.33 (-0.47 to -0.19) |
| Hattingh – 2009 | -0.32 (-0.46 to -0.18) |
| Isezuo – 2009 | -0.28 (-0.41 to -0.15) |
| Kaplan – 2000 | -0.33 (-0.46 to -0.19) |
| Kawai – 2011 | -0.34 (-0.47 to -0.20) |
| Kelly – 2002 | -0.32 (-0.46 to -0.19) |
| Kruger – 2005 | -0.32 (-0.46 to -0.18) |
| Longenecker – 2010 | -0.33 (-0.46 to -0.19) |
| Masaisa – 2011 | -0.32 (-0.46 to -0.19) |
| Mekonen – 2010 | -0.33 (-0.46 to -0.19) |
| Mercier – 2009 | -0.31 (-0.45 to -0.17) |
| Moore – 1993 | -0.33 (-0.46 to -0.19) |
| Mukaya – 2009 | -0.32 (-0.45 to -0.18) |
| Mutimura – 2010 | -0.34 (-0.47 to -0.20) |
| Niyongabo – 1999 | -0.31 (-0.44 to -0.17) |
| Noeske – 2006 | -0.33 (-0.47 to -0.19) |
| Noeske – 2011 | -0.32 (-0.45 to -0.18) |
| Okeahialam – 2006 | -0.32 (-0.46 to -0.18) |
| Papathakis – 2005 | -0.33 (-0.47 to -0.20) |
| Papathakis – 2006 | -0.32 (-0.46 to -0.19) |
| Praygod – 2011 | -0.32 (-0.46 to -0.18) |
| Range – 2010 | -0.33 (-0.47 to -0.19) |
| Sani – 2005 | -0.32 (-0.46 to -0.18) |
| Schutte – Unpublished | -0.32 (-0.46 to -0.18) |
| Sliwa – 2011 | -0.33 (-0.47 to -0.20) |
| Thompson – 2011 | -0.32 (-0.45 to -0.18) |
| Vorster – 2004 | -0.33 (-0.47 to -0.19) |
| Wallis – 2004 | -0.33 (-0.47 to -0.20) |
| MRC/UVRI - Unpublished | -0.33 (-0.47 to -0.19) |

- - =Unadjusted estimate, with no study excluded; SMD=Standardised mean difference; CI=Confidence interval; BMI=Body mass index

*Supplementary Table 5:* Sensitivity analysis of the change in combined standardised mean difference estimates for the association between HIV and TGs after sequential exclusion of single studies

| Excluded study | Combined SMD (95% CI) |
| --- | --- |
| - - | 0.26 (0.08 to 0.44) |
| Adewole – 2010 | 0.27 (0.08 to 0.46) |
| Becker – 2011 | 0.25 (0.07 to 0.43) |
| Erkistrup – 2007 | 0.26 (0.07 to 0.44) |
| Hattingh – 2009 | 0.30 (0.11 to 0.48) |
| Isezuo – 2009 | 0.22 (0.05 to 0.40) |
| Kruger – 2005 | 0.29 (0.09 to 0.48) |
| Lazar – 2009 | 0.25 (0.06 to 0.44) |
| Mercier – 2009 | 0.27 (0.08 to 0.46) |
| Mutimura – 2010 | 0.27 (0.08 to 0.47) |
| Nguemaim – 2010 | 0.16 (0.03 to 0.29) |
| Sani – 2005 | 0.26 (0.08 to 0.45) |
| Schutte – Unpublished | 0.30 (0.11 to 0.48) |
| Thompson – 2011 | 0.23 (0.05 to 0.41) |
| Vorster – 2004 | 0.30 (0.12 to 0.48) |
| MRC/UVRI - Unpublished | 0.27 (0.06 to 0.49) |

- - =Unadjusted estimate, with no study excluded; SMD=Standardised mean difference; CI=Confidence interval; TGs=Triglycerides

*Supplementary Table 6:* Sensitivity analysis of the change in combined standardised mean difference estimates for the association between HIV and LDL after sequential exclusion of single studies

| Excluded study | Combined SMD (95% CI) |
| --- | --- |
| - - | -0.16 (-0.34 to 0.03) |
| **Adewole – 2010** | **-0.27 (-0.39 to -0.14)** |
| Becker – 2011 | -0.12 (-0.31 to 0.06) |
| Erkistrup – 2007 | -0.12 (-0.31 to 0.07) |
| Isezuo – 2009 | -0.15 (-0.34 to 0.04) |
| Kruger – 2005 | -0.14 (-0.35 to 0.07) |
| Lazar – 2009 | -0.16 (-0.36 to 0.03) |
| Mercier – 2009 | -0.16 (-0.35 to 0.04) |
| Mutimura – 2010 | -0.16 (-0.36 to 0.04) |
| Nguemaim – 2010 | -0.13 (-0.33 to 0.06) |
| Sani – 2005 | -0.19 (-0.37 to 0.00) |
| Schutte – Unpublished | -0.11 (-0.29 to 0.07) |
| Thompson – 2011 | -0.15 (-0.34 to 0.04) |
| Vorster – 2004 | -0.15 (-0.36 to 0.05) |
| MRC/UVRI - Unpublished | -0.15 (-0.37 to 0.07) |

- - =Unadjusted estimate, with no study excluded; Bolded studies indicate a change in the interpretation of the combined SMD after exclusion of the study in question; SMD=Standardised mean difference; CI=Confidence interval; LDL=Low density lipoprotein cholesterol

*Supplementary Table 7:* Sensitivity analysis of the change in combined standardised mean difference estimates for the association between HIV and HDL after sequential exclusion of single studies

| Excluded study | Combined SMD (95% CI) |
| --- | --- |
| - - | -0.59 (-0.86 to -0.31) |
| Adewole – 2010 | -0.44 (-0.68 to -0.20) |
| Becker – 2011 | -0.57 (-0.85 to -0.28) |
| Erkistrup – 2007 | -0.54 (-0.82 to -0.26) |
| Isezuo – 2009 | -0.63 (-0.91 to -0.34) |
| Kruger – 2005 | -0.59 (-0.90 to -0.28) |
| Lazar – 2009 | -0.58 (-0.87 to -0.29) |
| Mercier – 2009 | -0.65 (-0.93 to -0.37) |
| Mutimura – 2010 | -0.59 (-0.89 to -0.29) |
| Nguemaim – 2010 | -0.52 (-0.78 to -0.26) |
| Sani – 2005 | -0.60 (-0.89 to -0.31) |
| Schutte – Unpublished | -0.61 (-0.91 to -0.31) |
| Thompson – 2011 | -0.63 (-0.92 to -0.35) |
| Vorster – 2004 | -0.61 (-0.92 to -0.30) |
| MRC/UVRI - Unpublished | -0.64 (-0.92 to -0.35) |

- - =Unadjusted estimate, with no study excluded; SMD=Standardised mean difference; CI=Confidence interval; HDL=High density lipoprotein cholesterol

*Supplementary Table 8:* Sensitivity analysis of the change in combined standardised mean difference estimates for the association between HIV and SBP after sequential exclusion of single studies

| Excluded study | Combined SMD (95% CI) |
| --- | --- |
| - - | -0.40 (-0.55 to -0.25) |
| Adewole – 2010 | -0.42 (-0.58 to -0.26) |
| Agaba – 2003 | -0.39 (-0.55 to -0.23) |
| Becker – 2011 | -0.40 (-0.56 to -0.24) |
| Compostella – 2008 | -0.39 (-0.54 to -0.23) |
| Hattingh – 2009 | -0.37 (-0.52 to -0.22) |
| Kaplan – 2000 | -0.37 (-0.53 to -0.22) |
| Kruger – 2005 | -0.40 (-0.57 to -0.23) |
| Lazar – 2009 | -0.41 (-0.58 to -0.25) |
| Longenecker – 2010 | -0.41 (-0.57 to -0.25) |
| Okeahialam – 2006 | -0.37 (-0.52 to -0.21) |
| Sani – 2005 | -0.37 (-0.52 to -0.21) |
| Schutte – Unpublished | -0.44 (-0.59 to -0.29) |
| Sliwa – 2011 | -0.42 (-0.58 to -0.26) |
| Vorster – 2004 | -0.43 (-0.59 to -0.26) |
| MRC/UVRI - Unpublished | -0.42 (-0.60 to -0.25) |

- - =Unadjusted estimate, with no study excluded; SMD=Standardised mean difference; CI=Confidence interval; SBP=Systolic blood pressure

*Supplementary Table 9:* Sensitivity analysis of the change in combined standardised mean difference estimates for the association between HIV and DBP after sequential exclusion of single studies

| Excluded study | Combined SMD (95% CI) |
| --- | --- |
| - - | -0.34 (-0.51 to -0.17) |
| Adewole – 2010 | -0.26 (-0.40 to -0.11) |
| Agaba – 2003 | -0.31 (-0.48 to -0.14) |
| Becker – 2011 | -0.35 (-0.53 to -0.17) |
| Compostella – 2008 | -0.32 (-0.49 to -0.15) |
| Hattingh – 2009 | -0.34 (-0.53 to -0.16) |
| Kaplan – 2000 | -0.32 (-0.49 to -0.15) |
| Kruger – 2005 | -0.35 (-0.54 to -0.15) |
| Lazar – 2009 | -0.34 (-0.52 to -0.16) |
| Longenecker – 2010 | -0.35 (-0.53 to -0.17) |
| Okeahialam – 2006 | -0.32 (-0.50 to -0.15) |
| Sani – 2005 | -0.33 (-0.50 to -0.15) |
| Schutte – Unpublished | -0.39 (-0.56 to -0.21) |
| Sliwa – 2011 | -0.37 (-0.54 to -0.19) |
| Vorster – 2004 | -0.37 (-0.56 to -0.18) |
| MRC/UVRI - Unpublished | -0.37 (-0.56 to -0.19) |

- - =Unadjusted estimate, with no study excluded; SMD=Standardised mean difference; CI=Confidence interval; DBP=Diastolic blood pressure

*Supplementary Table 10:* Sensitivity analysis of the change in combined standardised mean difference estimates for the association between HIV and glucose after sequential exclusion of single studies

| Excluded study | Combined SMD (95% CI) |
| --- | --- |
| - - | 0.35 (-0.35 to 1.06) |
| Hattingh – 2009 | 0.44 (-0.47 to 1.36) |
| Kruger – 2005 | 0.46 (-0.52 to 1.44) |
| Lazar – 2009 | 0.44 (-0.40 to 1.27) |
| **Mercier – 2009** | **-0.14 (-0.26 to -0.02)** |
| Schutte – Unpublished | 0.50 (-0.36 to 1.36) |
| Thompson – 2011 | 0.40 (-0.40 to 1.20) |

- - =Unadjusted estimate, with no study excluded; Bolded studies indicate a change in the interpretation of the combined SMD after exclusion of the study in question; SMD=Standardised mean difference; CI=Confidence interval

*Supplementary Table 11:* Sensitivity analysis of the change in combined standardised mean difference estimates for the association between HIV and HbA1c after sequential exclusion of single studies

| Excluded study | Combined SMD (95% CI) |
| --- | --- |
| - - | -0.07 (-0.39 to 0.25) |
| Fourie – Unpublished | 0.04 (-0.05 to 0.12) |
| Perret – 2000 | -0.16 (-0.54 to 0.23) |
| MRC/UVRI – Unpublished | -0.10 (-0.71 to 0.51) |

- - =Unadjusted estimate, with no study excluded; SMD=Standardised mean difference; CI=Confidence interval; HbA1c=Glycated hemoglobin

*Supplementary Figure 28:* Meta-analysis of the association between ART use and BMI

SMD=Standardised mean difference; CI=Confidence interval; SD=Standard deviation; BMI=Body mass index; ART=Antiretroviral therapy

*Supplementary Figure 29:* Meta-analysis of the association between ART use and TGs

SMD=Standardised mean difference; CI=Confidence interval; SD=Standard deviation; TGs=Triglycerides; ART=Antiretroviral therapy

*Supplementary Figure 30:* Meta-analysis of the association between ART use and LDL

SMD=Standardised mean difference; CI=Confidence interval; SD=Standard deviation; LDL=Low density lipoprotein cholesterol; ART=Antiretroviral therapy

*Supplementary Figure 31:* Meta-analysis of the association between ART use and HDL

SMD=Standardised mean difference; CI=Confidence interval; SD=Standard deviation; HDL=High density lipoprotein cholesterol; ART=Antiretroviral therapy

*Supplementary Figure 32:* Meta-analysis of the association between ART use and SBP

SMD=Standardised mean difference; CI=Confidence interval; SD=Standard deviation; SBP=Systolic blood pressure; ART=Antiretroviral therapy

*Supplementary Figure 33:* Meta-analysis of the association between ART use and DBP

SMD=Standardised mean difference; CI=Confidence interval; SD=Standard deviation; DBP=Diastolic blood pressure; ART=Antiretroviral therapy

*Supplementary Figure 34:* Meta-analysis of the association between ART use and glucose

SMD=Standardised mean difference; CI=Confidence interval; SD=Standard deviation; ART=Antiretroviral therapy

*Supplementary Figure 35:* Meta-analysis of the association between ART use and HbA1c

SMD=Standardised mean difference; CI=Confidence interval; SD=Standard deviation; ART=Antiretroviral therapy; HbA1c=Glycated hemoglobin

*Supplementary Figure 36:* Summary of overall estimates from fixed effects meta-analyses of ART associations with individual cardiometabolic traits

SMD=Standardised mean difference; CI=Confidence interval; BMI=Body mass index; TGs=Triglycerides; LDL=Low density lipoprotein cholesterol; HDL=High density lipoprotein cholesterol; SBP=Systolic blood pressure; DBP=Diastolic blood pressure; ART=Antiretroviral therapy; HbA1c=Glycated hemoglobin

*Supplementary Figure 37:* Stratified analysis of heterogeneity of the association between ART and BMI

SMD=Standardised mean difference; CI=Confidence interval; BMI=Body mass index; ART=Antiretroviral therapy

*Supplementary Figure 38:* Stratified analysis of heterogeneity of the association between ART and TGs

SMD=Standardised mean difference; CI=Confidence interval; TGs=Triglycerides; ART=Antiretroviral therapy

*Supplementary Figure 39:* Stratified analysis of heterogeneity of the association between ART and LDL

SMD=Standardised mean difference; CI=Confidence interval; LDL=Low density lipoprotein cholesterol; ART=Antiretroviral therapy

*Supplementary Figure 40:* Stratified analysis of heterogeneity of the association between ART and HDL

SMD=Standardised mean difference; CI=Confidence interval; HDL=High density lipoprotein cholesterol; ART=Antiretroviral therapy

*Supplementary Figure 41:* Stratified analysis of heterogeneity of the association between ART and SBP

SMD=Standardised mean difference; CI=Confidence interval; SBP=Systolic blood pressure; ART=Antiretroviral therapy

*Supplementary Figure 42:* Stratified analysis of heterogeneity of the association between ART and DBP

SMD=Standardised mean difference; CI=Confidence interval; DBP=Diastolic blood pressure; ART=Antiretroviral therapy

*Supplementary Figure 43:* Stratified analysis of heterogeneity of the association between ART and glucose

SMD=Standardised mean difference; CI=Confidence interval; ART=Antiretroviral therapy

*Supplementary Figure 44:* Stratified analysis of heterogeneity of the association between ART and HbA1c

SMD=Standardised mean difference; CI=Confidence interval; ART=Antiretroviral therapy; HbA1c=Glycated hemoglobin

*Supplementary Figure 45:* Galbraith plot of the association between ART and BMI

Dots represent studies. Unweighted regression line with 95% confidence intervals superimposed as three parallels lines. In the absence of heterogeneity, we would expect all studies to lie within the 95% confidence intervals; b=Standardised mean difference estimate; se=Standard error for each estimate (b); BMI=Body mass index

*Supplementary Figure 46:* Galbraith plot of the association between ART and TGs

Dots represent studies. Unweighted regression line with 95% confidence intervals superimposed as three parallels lines. In the absence of heterogeneity, we would expect all studies to lie within the 95% confidence intervals; b=Standardised mean difference estimate; se=Standard error for each estimate (b); TGs=Triglycerides

*Supplementary Figure 47:* Galbraith plot of the association between ART and LDL

Dots represent studies. Unweighted regression line with 95% confidence intervals superimposed as three parallels lines. In the absence of heterogeneity, we would expect all studies to lie within the 95% confidence intervals; b=Standardised mean difference estimate; se=Standard error for each estimate (b); LDL=Low density lipoprotein cholesterol

*Supplementary Figure 48:* Galbraith plot of the association between ART and HDL

Dots represent studies. Unweighted regression line with 95% confidence intervals superimposed as three parallels lines. In the absence of heterogeneity, we would expect all studies to lie within the 95% confidence intervals; b=Standardised mean difference estimate; se=Standard error for each estimate (b); HDL=High density lipoprotein cholesterol

*Supplementary Figure 49:* Galbraith plot of the association between ART and SBP

Dots represent studies. Unweighted regression line with 95% confidence intervals superimposed as three parallels lines. In the absence of heterogeneity, we would expect all studies to lie within the 95% confidence intervals; b=Standardised mean difference estimate; se=Standard error for each estimate (b); SBP=Systolic blood pressure

*Supplementary Figure 50:* Galbraith plot of the association between ART and DBP

Dots represent studies. Unweighted regression line with 95% confidence intervals superimposed as three parallels lines. In the absence of heterogeneity, we would expect all studies to lie within the 95% confidence intervals; b=Standardised mean difference estimate; se=Standard error for each estimate (b); DBP=Diastolic blood pressure

*Supplementary Figure 51:* Galbraith plot of the association between ART and glucose

Dots represent studies. Unweighted regression line with 95% confidence intervals superimposed as three parallels lines. In the absence of heterogeneity, we would expect all studies to lie within the 95% confidence intervals; b=Standardised mean difference estimate; se=Standard error for each estimate (b)

*Supplementary Figure 52:* Galbraith plot of the association between ART and HbA1c

Dots represent studies. Unweighted regression line with 95% confidence intervals superimposed as three parallels lines. In the absence of heterogeneity, we would expect all studies to lie within the 95% confidence intervals; b=Standardised mean difference estimate; se=Standard error for each estimate (b); HbA1c=Glycated hemoglobin

*Supplementary Table 12:* Sensitivity analysis of the change in combined standardised mean difference estimates for the association between ART and BMI after sequential exclusion of single studies

| Excluded study | Combined SMD (95% CI) |
| --- | --- |
| - - | 0.12 (-0.11 to 0.34) |
| Adewole – 2010 | 0.02 (-0.10 to 0.13) |
| Ahoua – 2011 | 0.13 (-0.13 to 0.38) |
| Awotedu – 2010 | 0.12 (-0.12 to 0.36) |
| Dave – 2011 | 0.11 (-0.15 to 0.37) |
| Fourie – Unpublished | 0.15 (-0.09 to 0.38) |
| Ngondi – 2007 | 0.15 (-0.09 to 0.38) |
| Nzou – 2010 | 0.14 (-0.10 to 0.37) |
| Pefura – 2011 | 0.09 (-0.15 to 0.33) |
| Praygod – 2011 | 0.12 (-0.12 to 0.37) |
| Sani – 2005 | 0.12 (-0.12 to 0.36) |
| Scarcella – 2011 | 0.15 (-0.09 to 0.38) |
| Thompson – 2011 | 0.10 (-0.14 to 0.34) |
| MRC/UVRI – Unpublished | 0.13 (-0.11 to 0.38) |

- - =Unadjusted estimate, with no study excluded; SMD=Standardised mean difference; CI=Confidence interval; BMI=Body mass index; ART=Antiretroviral therapy

*Supplementary Table 13:* Sensitivity analysis of the change in combined standardised mean difference estimates for the association between ART and TGs after sequential exclusion of single studies

| Excluded study | Combined SMD (95% CI) |
| --- | --- |
| - - | 0.09 (-0.04 to 0.21) |
| Adewole – 2010 | 0.10 (-0.04 to 0.24) |
| Buchacz – 2008 | 0.11 (-0.03 to 0.25) |
| Dave – 2011 | 0.08 (-0.07 to 0.22) |
| Fourie – Unpublished | 0.05 (-0.07 to 0.16) |
| Ngondi – 2007 | 0.08 (-0.06 to 0.21) |
| Ogundahunsi – 2008 | 0.07 (-0.06 to 0.20) |
| Pefura – 2011 | 0.09 (-0.06 to 0.23) |
| Sani – 2005 | 0.08 (-0.06 to 0.22) |
| **Thompson – 2011** | **0.12 (0.00 to 0.24)** |
| MRC/UVRI – Unpublished | 0.11 (-0.03 to 0.25) |

- - =Unadjusted estimate, with no study excluded; Bolded studies indicate a change in the interpretation of the combined SMD after exclusion of the study in question; SMD=Standardised mean difference; CI=Confidence interval; TGs=Triglycerides; ART=Antiretroviral therapy

*Supplementary Table 14:* Sensitivity analysis of the change in combined standardised mean difference estimates for the association between ART and LDL after sequential exclusion of single studies

| Excluded study | Combined SMD (95% CI) |
| --- | --- |
| - - | 0.43 (0.14 to 0.72) |
| Adewole – 2010 | 0.53 (0.29 to 0.77) |
| Buchacz – 2008 | 0.34 (0.10 to 0.58) |
| Dave – 2011 | 0.41 (0.06 to 0.76) |
| Fourie – Unpublished | 0.41 (0.09 to 0.72) |
| Ngondi – 2007 | 0.38 (0.08 to 0.69) |
| Ogundahunsi – 2008 | 0.40 (0.09 to 0.71) |
| Pefura – 2011 | 0.43 (0.11 to 0.75) |
| Sani – 2005 | 0.44 (0.13 to 0.76) |
| Thompson – 2011 | 0.47 (0.17 to 0.77) |
| MRC/UVRI – Unpublished | 0.45 (0.13 to 0.77) |

- - =Unadjusted estimate, with no study excluded; SMD=Standardised mean difference; CI=Confidence interval; LDL=Low density lipoprotein cholesterol; ART=Antiretroviral therapy

*Supplementary Table 15:* Sensitivity analysis of the change in combined standardised mean difference estimates for the association between ART and HDL after sequential exclusion of single studies

| Excluded study | Combined SMD (95% CI) |
| --- | --- |
| - - | 0.39 (0.11 to 0.66) |
| Adewole – 2010 | 0.32 (0.02 to 0.62) |
| Buchacz – 2008 | 0.31 (0.03 to 0.58) |
| Dave – 2011 | 0.35 (0.01 to 0.68) |
| Fourie – Unpublished | 0.41 (0.11 to 0.70) |
| Ngondi – 2007 | 0.45 (0.18 to 0.73) |
| Ogundahunsi – 2008 | 0.49 (0.23 to 0.74) |
| Pefura – 2011 | 0.43 (0.15 to 0.71) |
| Sani – 2005 | 0.38 (0.08 to 0.69) |
| Thompson – 2011 | 0.37 (0.07 to 0.67) |
| MRC/UVRI – Unpublished | 0.35 (0.02 to 0.68) |

- - =Unadjusted estimate, with no study excluded; SMD=Standardised mean difference; CI=Confidence interval; HDL=High density lipoprotein cholesterol; ART=Antiretroviral therapy

*Supplementary Table 16:* Sensitivity analysis of the change in combined standardised mean difference estimates for the association between ART and SBP after sequential exclusion of single studies

| Excluded study | Combined SMD (95% CI) |
| --- | --- |
| - - | 0.05 (-0.19 to 0.28) |
| Adewole – 2010 | 0.16 (-0.01 to 0.33) |
| Dave – 2011 | -0.03 (-0.27 to 0.21) |
| Fourie – Unpublished | 0.03 (-0.24 to 0.30) |
| Nzou – 2010 | 0.09 (-0.16 to 0.34) |
| Sani – 2005 | 0.04 (-0.24 to 0.32) |
| MRC/UVRI - Unpublished | 0.00 (-0.31 to 0.31) |

- - =Unadjusted estimate, with no study excluded; SMD=Standardised mean difference; CI=Confidence interval; SBP=Systolic blood pressure; ART=Antiretroviral therapy

*Supplementary Table 17:* Sensitivity analysis of the change in combined standardised mean difference estimates for the association between ART and DBP after sequential exclusion of single studies

| Excluded study | Combined SMD (95% CI) |
| --- | --- |
| - - | 0.06 (-0.10 to 0.22) |
| **Adewole – 2010** | **0.16 (0.06 to 0.26)** |
| Dave – 2011 | 0.00 (-0.17 to 0.17) |
| Fourie – Unpublished | 0.07 (-0.11 to 0.25) |
| Nzou – 2010 | 0.07 (-0.10 to 0.25) |
| Sani – 2005 | 0.03 (-0.16 to 0.23) |
| MRC/UVRI - Unpublished | 0.03 (-0.19 to 0.24) |

- - =Unadjusted estimate, with no study excluded; Bolded studies indicate a change in the interpretation of the combined SMD after exclusion of the study in question; SMD=Standardised mean difference; CI=Confidence interval; DBP=Diastolic blood pressure; ART=Antiretroviral therapy

*Supplementary Table 18:* Sensitivity analysis of the change in combined standardised mean difference estimates for the association between ART and glucose after sequential exclusion of single studies

| Excluded study | Combined SMD (95% CI) |
| --- | --- |
| - - | -0.23 (-0.61 to 0.16) |
| Dave – 2011 | -0.34 (-0.78 to 0.09) |
| Fourie – Unpublished | -0.29 (-0.77 to 0.20) |
| Ngondi – 2007 | -0.04 (-0.30 to 0.22) |
| Sani – 2005 | -0.30 (-0.81 to 0.21) |
| Thompson – 2011 | -0.17 (-0.61 to 0.26) |

- - =Unadjusted estimate, with no study excluded; SMD=Standardised mean difference; CI=Confidence interval; ART=Antiretroviral therapy

*Supplementary Table 19* Sensitivity analysis of the change in combined standardised mean difference estimates for the association between ART and HbA1c after sequential exclusion of single studies

| Excluded study | Combined SMD (95% CI) |
| --- | --- |
| - - | -0.34 (-0.62 to -0.06) |
| Fourie – Unpublished | -0.34 (-0.40 to -0.06) |
| MRC/UVRI – Unpublished | -0.52 (-0.86 to -0.19) |

- - =Unadjusted estimate, with no study excluded; SMD=Standardised mean difference; CI=Confidence interval; ART=Antiretroviral therapy; HbA1c=Glycated hemoglobin
